# Environmental DNA reveals quantitative patterns of fish biodiversity in large rivers despite its downstream transportation

**DOI:** 10.1038/s41598-018-28424-8

**Published:** 2018-07-10

**Authors:** Didier Pont, Mathieu Rocle, Alice Valentini, Raphaël Civade, Pauline Jean, Anthony Maire, Nicolas Roset, Michael Schabuss, Horst Zornig, Tony Dejean

**Affiliations:** 1 SPYGEN, 17 rue du Lac Saint-André, Savoie Technolac, 73370 Le Bourget du Lac, France; 2 VIGILIFE, 17 rue du Lac Saint-André, Savoie Technolac, 73370 Le Bourget du Lac, France; 3 Institute of Hydrobiology and Aquatic Ecosystem Management (IHG), University of Natural Resources and Life Sciences, Gregor-Mendel-Strasse 33, 1180 Vienna, Austria; 4 Compagnie Nationale du Rhône, Direction de l’Ingénierie, 2 rue André Bonin, 69004 Lyon, France; 5 EDF R&D, LNHE (Laboratoire National d’Hydraulique et Environnement), 6 quai Watier, 78401 Chatou Cedex, France; 6 French National Agency for Biodiversity, Auvergne-Rhone-Alpes Regional Directorate, Parc de Parilly, Chemin des Chasseurs, 69500 Bron, France; 7 PRO FISCH OG Ecological Consultants, Hörlgasse 6, A-1090 Vienna, Austria

## Abstract

Despite the ecological and societal importance of large rivers, fish sampling remains costly and limited to specific habitats (e.g., river banks). Using an eDNA metabarcoding approach, we regularly sampled 500 km of a large river (Rhône River). Comparisons with long-term electrofishing surveys demonstrated the ability of eDNA metabarcoding to qualitatively and quantitatively reveal fish assemblage structures (relative species abundance) but eDNA integrated a larger space than the classical sampling location. Combination of a literature review and field data showed that eDNA behaves in the water column like fine particulate organic matter. Its detection distance varied from a few km in a small stream to more than 100 km in a large river. To our knowledge, our results are the first demonstration of the capacity of eDNA metabarcoding to describe longitudinal fish assemblage patterns in a large river, and metabarcoding appears to be a reliable, cost-effective method for future monitoring.

## Introduction

As the impacts of human activities on our planet continue to increase^1^, all ecosystems are profoundly altered. Aquatic systems, particularly rivers, are recognized as being highly degraded in terms of both species diversity and ecological functioning^2^. The rate of decline in aquatic populations is almost twice that of terrestrial populations^3^. Facing this dramatic development, increasing the scale and frequency of aquatic biodiversity observations is a priority^4^. Among other bioindicators, fish are substantially valuable to water managers and the public. However, due to sampling difficulties, the probability of detecting rare species is low^5^, and quantitatively monitoring fish assemblages remains a hard and costly task, particularly in large rivers^6^.

Since its first application to macroorganisms^7^, environmental DNA (eDNA) has increasingly appeared to be a promising non-invasive method for improving aquatic biodiversity monitoring^8^. eDNA refers to DNA obtained from environmental samples without the prior isolation of any target organism^9^. In the case of water samples, eDNA contains both intra-organism DNA (e.g., small planktonic organisms) and extra-organism DNA (e.g., from fish) which can be cellular or extracellular and degraded^9^. While DNA can persist for long periods in dry, cold conditions and in the absence of light^7^, eDNA persistence in water and normal temperature conditions varies from a few days to a few weeks^10–12^. With the emergence of next-generation sequencing (NGS) platforms and the use of universal PCR primers (eDNA metabarcoding), large collections of taxa can be identified via a single experiment^13^. This not only offers the possibility to detect rare or evasive species^8^ without a priori but also allows the rapid biodiversity assessment of large communities and the reconstruction of ecological and evolutionary processes from easy-to-collect samples^14^.

Previous studies have shown that this approach is effective for inventorying fish and amphibian species in mesocosms^15,16^, estuarine and marine systems^17–19^, lakes^20,21^, rivers and ponds^22–24^. However, like other inventory methods, eDNA metabarcoding has its own biases. False-negative detections can occur, which are mainly due to failure of the method (low marker sensitivity, low eDNA quantity, ineffective sample preservation^25^, PCR inhibitors^26^ and/or insufficient sampling efforts^27^). False-positive detections can be related to other failures of the method (e.g., contamination and lack of DNA marker species specificity) as well as to the contamination of the studied system by external sources of eDNA, such as sewage effluents and animal excrements^25,28^.

In streams, the concentration of eDNA and its detectability are not only dependent on production and degradation rates but also on dilution, transport through the river network, deposition and resuspension^27^. The observed eDNA detection distances vary between studies from less than one km in mesocosm and field experiments^27,29^ to around ten km at the outlet of lakes when detecting eDNA from two lake-dwelling invertebrate species^30^. This last result suggests that eDNA in rivers integrates diversity information over space on the catchment scale^31^. In a similar study, the detection distance of eDNA from a lake-dwelling fish in the outlet stream was also high (up to 3.6 km)^22^. Nevertheless, this study also demonstrated that eDNA metabarcoding could reveal changes in fish communities along a small river stretch of less than 7 km, proving that this method can highlight biodiversity patterns along the river. Other studies have also demonstrated such spatial changes in fish communities along environmental gradients^19,28,32^. However, the methodological differences between these studies hamper the detailed comparison of their results. Nevertheless, the detection distance in rivers is a key point to analyze how eDNA can reveal spatial and temporal changes in biological community structures, and additional efforts are needed to understand the spatiotemporal dynamics of eDNA in aquatic systems^33^.

Another major challenge of eDNA methods is whether they enable the estimation of animal abundance^8,15,34^. Quantitative eDNA analyses (qPCR, ddPCR) allow investigations into the strength of the relationship between eDNA concentrations and the density or biomass of a single fish species in mesocosms^15,35,36^ or under natural conditions^25,29,37,38^. Nevertheless, the eDNA concentration often explains a moderate amount of fish abundance variability^39^ and is either better correlated with the number of individuals or the biomass. When using metabarcoding methods, quantitative fish detection is accomplished by assessing relationships between the abundance estimations obtained from traditional sampling methods and the number of eDNA copies or the frequency of positive samples^27^. The correlations proved to be significant, but their moderate strengths implied that these results are more of a first proof-of-concept rather than a demonstration of a quantitative relationship with fish stocks. Higher water temperatures improved this relationship^40^. In addition to the numerous factors influencing eDNA concentrations and dispersion in water, biases along the analytical pipeline can strongly alter the relationship between the initial quantity of eDNA in the water sample and the final number of reads per species^14,33,41^. Nevertheless, a recent experiment demonstrated a high correlation between the number of sequence reads and the relative amounts of MOTUs (metabarcoding operational taxonomic units) when primers were carefully designed^42^.

**Figure 1:**
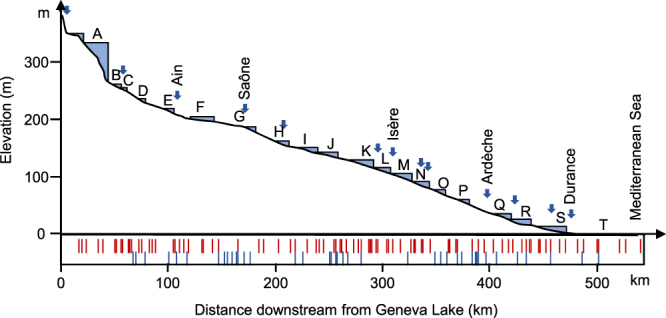
Longitudinal profile of the Rhône River from Lake Geneva to the Mediterranean Sea (540 km). Locations of the hydroelectric power schemes and river sections from A to T (i.e., A-GE to T-PA, see Supplementary Table 1 for the associated names). Locations of the confluence points with the main tributaries (and names of the five most important). Box: locations of the 59 eDNA samples (in red) and 40 TEF long-term surveys (in blue).

In this paper, we analyzed the capacity of eDNA metabarcoding to reveal quantitative patterns of fish biodiversity along a major European river, the Rhône River. eDNA was regularly sampled (Fig. 1) from Lake Geneva to the Mediterranean Sea (524 km long), and we used a metabarcoding approach previously described^21^. First, to evaluate the ability of number of reads of MOTUs to allow between sites comparison of species relative abundance, we investigated its relationship with the detection rate for each of the MOTUs. Second, by comparing our results with historical traditional electrofishing (TEF) surveys, we tested the ability of eDNA metabarcoding to i) estimate species richness and relative species abundances on the local scale (a few kilometers), and ii) to reveal quantitative patterns of fish biodiversity on the scale of the entire longitudinal course of the river. Third, by combining field data and previous knowledge related to the behavior of fine particulate organic matter (FPOM) in rivers, we simulated the detection distances of eDNA metabarcoding for a large range of hydraulic conditions. Finally, we herein discussed the possibility of using eDNA metabarcoding for river monitoring.

## Results

### Relation between detection rate and number of MOTU’s copies

**Figure 2:**
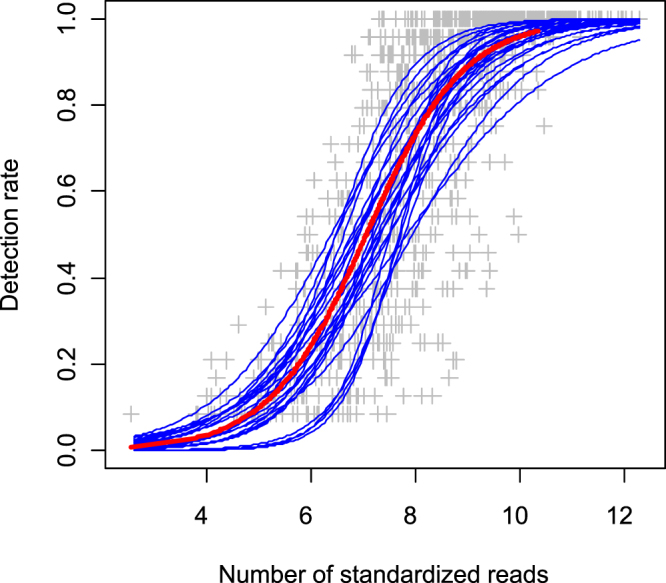
Relationship between number of standardized reads (in log) and detection rate (frequency of positive PCR) of MOTUs among sites. Grey cross: observed values. Red line: predicted detection rate as a function of the number of standardized reads (glm model). Blue lines: predicted detection rate as a function of the number of standardized reads, MOTU’s identity and their interaction (glm model). List of the 24 MOTUs considered in the second model: Abr_bra, Alb_alb, Alb_bip, Alo_spp, Ang_ang, Bar_bar, Bli_bjo, Cor_lav, Cot_sp, Cyp_car, Cypr_1, Eso_luc, Lep_gib, Leu_spp, Liz_ram, Onc_myk, Perc_flu, Pho_pho, Pse_par, Rho_ser, Sal_spp, Sal_tru San_luc, Sil_gla (see Supplementary Table 2 for the species names corresponding to the abbreviations).

When each of the 24 selected MOTUs (taxa) was modelled separately, all the relationships between number of standardized reads and detection rate were significant (explained deviance range: 32.3% to 87.6%, P < 10^−20^ for each species). When pooling all the MOTUs (Fig. 2), the global model was also highly significant (P < 10^−15^) and explained 64.4% of the total deviance. The detection rates were 5% (close to the detection rate for one positive PCR in 24), 50% (12 positive PCR in 24) and 95% (close to 23 positive PCR in 24) when MOTU’s relative abundance were respectively 0.02%, 0.30% and 5.49%. Adding MOTU’s identity and the interaction with the number of standardized reads to the model increased the explained deviance up to 70.6% of the total deviance (P < 10^−15^). MOTU’s identity and the interaction had both a significant effect (P < 10^−15^) and explained respectively 4.21% and 1.93% of the total deviance in addition to the deviance explained by the number of standardized reads (Fig. 2). For a predicted detection rate of 5%, 50% and 95% the relative abundance of MOTUs ranges were respectively 0.01–0.11% (median value: 0.02%), 0.15–0.71% (median value: 0.37%) and 1.56–25.68% (median value: 5.18%) with the last model.

### Comparison of eDNA metabarcoding and TEF samples on the sampling site scale

Among the 48 taxa detected by eDNA metabarcoding, four species likely do not exist in the Rhône River^43^. *Argyrosomus regius* and *Salmo salar* have never been recorded in the Rhône catchment. *Salvelinus_spp* includes cold water species inhabiting only lakes or small brooks, and *Oncorhynchus mykiss* is a farmed species^43^. The relative numbers of reads for *A*. *regius* and *S*. *salar* were low (maximums of 0.08 to 0.78%, respectively), but *O*. *mykiss* and *Salvelinus spp*. had normal read numbers (maximums of 5.0% and 89%, respectively). Among the other 44 species detected by eDNA, four were not registered in our TEF dataset but are known to be present in the Rhône River^43^ (*Chelon labrosus*, *Mugil cephalus*, *Zingel asper*, *Misgurnus fossilis*). Two species caught by TEF were also detected by eDNA, but the numbers of DNA copies were below our threshold, and the species were discarded (*Leuciscus delineatus*, *Lota lota*).

**Figure 3:**
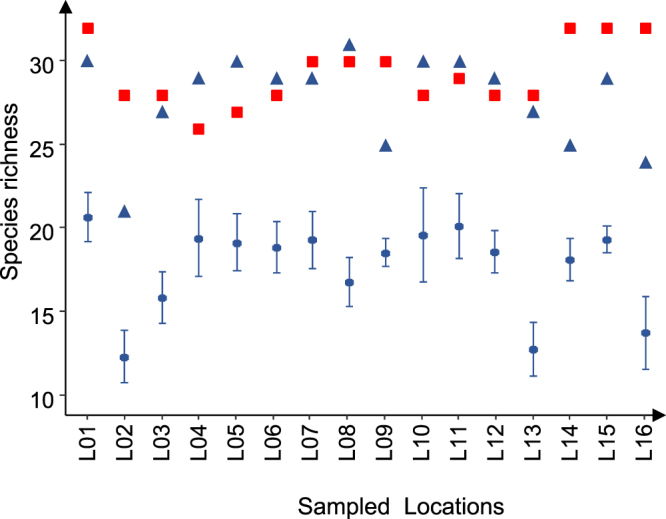
Comparison of the mean number of species (±95% confidence interval) caught annually with TEF (blue circle), the total number of species (blue triangle) caught during the 10-year survey (2006–2016), and the number of species detected in eDNA samples (red squares) for each of the 16 locations (L01 to L16) sampled with both methods (see Supplementary Table 1).

Of the 16 selected river locations (L1 to L16, see Supplementary Table 1) for which pairs of eDNA and TEF samples were compared, the mean species richness values of the annual TEF samples (12.3 to 20.6 species) were always significantly lower (P < 0.001) than those of the paired eDNA samples (26 to 33 species), i.e., 28.6% to 57.7% fewer species were identified in the TEF samples (Fig. 3). Considering the species that were sampled at least once during the ten years of the TEF surveys, the total (or cumulative) richness per sampling site (21 to 30 species) did not differ from that estimated by eDNA metabarcoding in one sampling session (Wilcoxon signed-rank test, P > 0.05). Furthermore, 73% to 93% of the total numbers of species detected in each river location were common to both the TEF and eDNA samples (Table 1). Zero to ten species per river location were detected by only eDNA, and they represented less than 1% of the total number of reads per sample in 84% of the cases (maximum of 4.1% of the total number of reads). The most frequent taxa not detected by TEF were Cypr_2, *Salmo trutta*, *Lampetra spp* and *Salaria fluviatilis*. Zero to five species per river location were detected by only TEF. The most frequent taxa not detected by eDNA metabarcoding were *Anguilla anguilla*, *Scardinius erythrophthalmus*, *Alosa spp* and *Leuciscus spp*. These taxa represented less than 1% of the total number of fish caught except in one case (*Ameiurus melas*: 2.66%).

**Table 1:** Comparison of species detections in eDNA and TEF samples (accumulated over ten years) at 16 sampling locations along the Rhône River.

Sampling locations	Nb. of species in common	Nb. of species in only eDNA metabarcoding data	Nb. of species in only TEF data	Spearman’s coefficient (p value)
L01	29	4	1	0.261 (p = 0.1714)
L02	20	9	1	0.653 (p = 0.0018)
L03	25	4	2	0.512 (p = 0.0088)
L04	25	1	4	0.45 (p = 0.0241)
L05	25	2	5	0.618 (p = 0.001)
L06	25	3	4	0.333 (p = 0.104)
L07	28	2	1	0.474 (p = 0.0107)
L08	28	2	3	0.814 (p < 0.0001)
L09	25	5	0	0.346 (p = 0.0905)
L10	28	0	2	0.643 (p = 0.0002)
L11	28	1	2	0.478 (p = 0.0101)
L12	26	2	3	0.578 (p = 0.002)
L13	24	4	3	0.594 (p = 0.0022)
L14	24	8	1	0.563 (p = 0.0042)
L15	26	6	3	0.726 (p < 0.0001)
L16	22	10	2	0.53 (p < 0.0001)

The numbers of species detected in both eDNA and TEF samples and in only one of the two methods are specified. The Spearman’s correlation coefficients between the standardized number of reads and the number of individuals caught per species and per CPUE (see Methods for details) and the associated p-values are also given.

The correlations between the number of reads (eDNA) and the number of fish caught (TEF) were significant (Spearman’s rank correlation test) in 13 of the 16 selected river locations (Table 1). For six species (*Barbus barbus*, *Abramis brama*, *Gymnocephalus cernuus*, *Barbatula barbatula*, *Phoxinus phoxinus*, *Sander lucioperca*), the relative abundance in the eDNA sample was significantly higher than that in the TEF sample (non-parametric signed test, P < 0.01 after Bonferroni correction for multiple comparisons). Only two species had significantly higher relative abundances in the TEF samples than in the eDNA samples: *Alburnus alburnus* (P < 0.001) and *Squalius cephalus* (P < 0.05).

**Figure 4:**
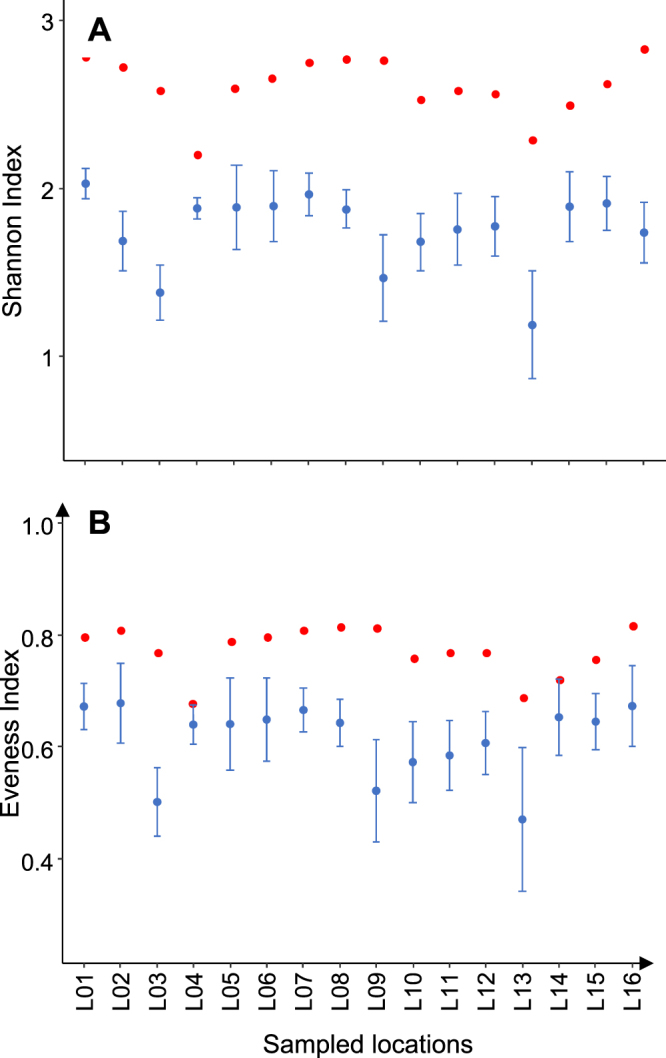
Mean values (±95% confidence interval) of the (**A**) Shannon and (**B**) Evenness indices for the 10 annual TEF samples (blue circles) and the eDNA samples collected in 2016 (red circles) at each of the 16 sampling locations (L01 to L16).

The Shannon and Evenness indices from the eDNA samples (2.21 to 2.83 and 0.68 to 0.82, respectively) were significantly higher than the mean Shannon (Wilcoxon signed-rank test, P < 0.001) and mean Evenness indices (Wilcoxon signed-rank test, P < 0.001 to P < 0.05) from the annual TEF samples (1.19 to 2.03 and 0.47 to 0.68, respectively) (Fig. 4).

### Longitudinal patterns of fish biodiversity revealed by eDNA metabarcoding and TEF

**Figure 5:**
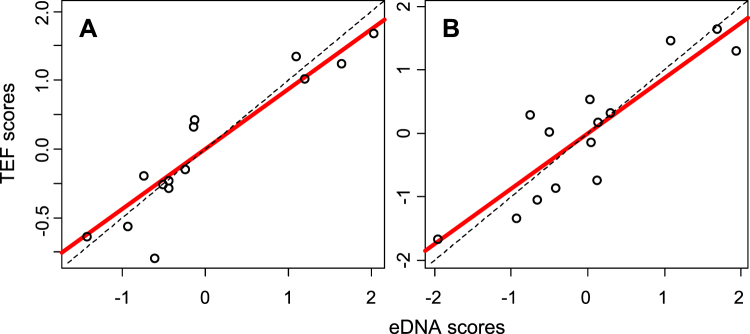
Relationships between the standardized scores of the eDNA and TEF samples for each of the 14 river sections on the first (**A**) and second (**B**) principle components of the co-inertia analysis. Solid line: regression of TEF samples on standardized scores of the eDNA samples. Dashed line: regression line with an intercept of zero and a slope of one (dashed line).

According to eigenvalues associated with the PCA analyses of eDNA (number of reads) and TEF samples from 14 of the 16 successive river sections from Lake Geneva to the sea, the first two principal components (PC1 and PC2) explained most of the structures for both datasets (59.1% and 51.8% of the total inertia, respectively). Regarding the co-inertia analysis performed using these first two principal components (PC), the co-inertia criterion was highly significant (RV = 0.736, p < 0.0001), which demonstrates a clear similarity between the fish assemblage structures described by eDNA metabarcoding and TEF in each of the 14 river sections. The first two co-inertia axes accounted for 85.4% of the total inertia. The first and second co-inertia PCs were highly correlated with the first and second PCs of the independent PCAs performed on the eDNA metabarcoding (R = −0.982 and R = −0.951, respectively) and TEF (R = −0.923 and R = −0.872, respectively) datasets. The coordinates of the eDNA and TEF samples on the first and second co-inertia PCs (Fig. 5) were highly correlated (R^2^ = 0.859 and R^2^ = 0.763, respectively, P < 0.001). The slope and intercept of the two regression lines did not differ from zero and one, respectively (Student’s t-test, P > 0.05).

**Figure 6:**
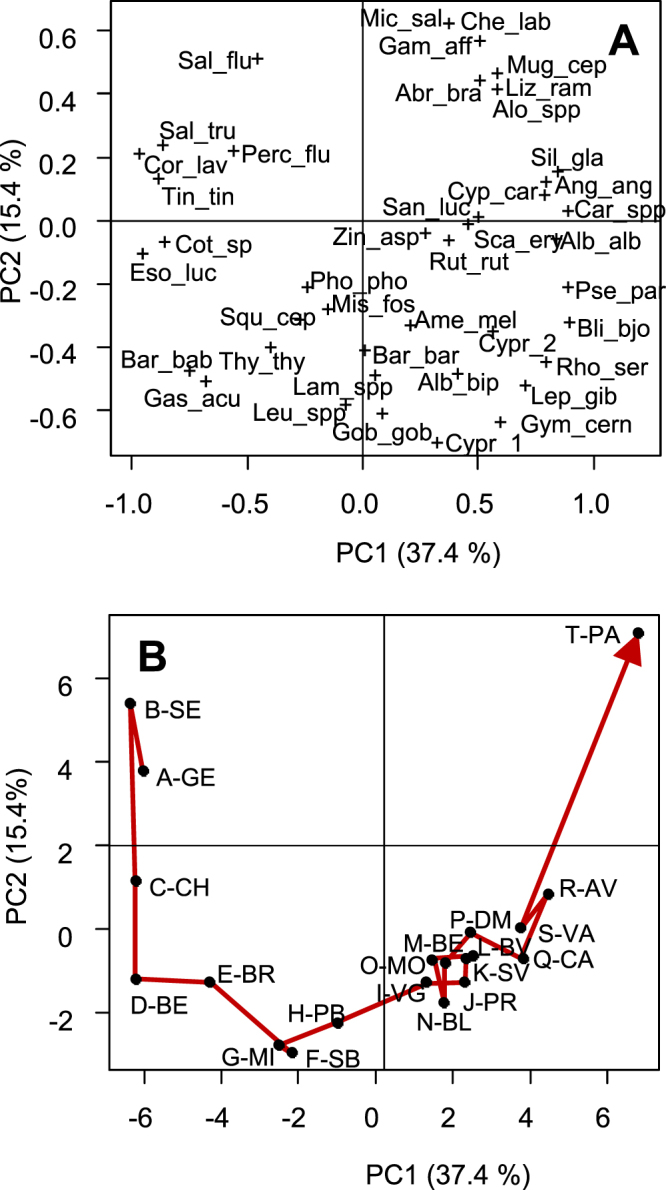
Longitudinal profile of fish diversity as determined by principal component analysis performed on the eDNA samples from the Rhône River. Scores of the 42 species (**A**) and 20 successive river sections (**B**) on the two first principal components, PC1 and PC2 (See Supplementary Table 1 for the names of the successive 20 river sections (A-GE to T-PA) and Supplementary Table 2 for the species names corresponding to the abbreviations).

The first and the second principal components (PC1 and PC2) of the PCAs performed on all the pooled eDNA samples within each of the 20 successive river sections accounted for 37.4 and 15.4%, respectively, of the total inertia of the dataset (Fig. 6). Among the 42 species studied, 15 and 16 species had a loading >3% on PC1 and PC2, respectively. PC1 ordered the sections from upstream to downstream, and PC2 differentiated the first two river sections (A-GE, B-SE, large impoundments), associated with typical lake species (*Coregonus lavaretus*, *tinca*, *Perca fluviatilis*), and the last deltaic river section (T-PA), associated with estuarine species (*C*. *labrosus*, *Liza ramada*, *Mugil cephalus*). The river sections from the upper Rhône (C-CH to G-MI) were mainly characterized by lotic species (*B*. *barbus*, *Leuciscus spp*, *Thymallus*, *Cottus sp*.). Downstream from confluence with the Saône River, the river sections (I-VG to P-DM) had similar scores on both axes, and river sections Q-CA through R-AV were associated with typical lentic species (*Silurus glanis*, *Cyprinus carpio*, *Carassius spp*.) and long-distance migratory species (*A*. *anguilla*, *Alosa spp*).

**Figure 7:**
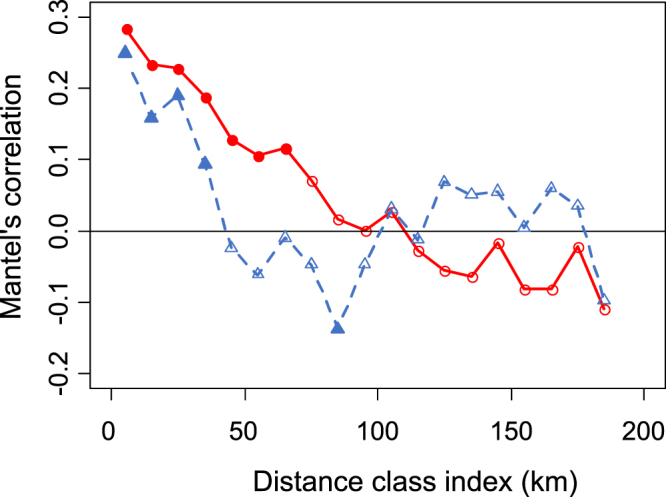
Mantel correlation between dissimilarities in fish species compositions (Bray-Curtis β-diversity) and distances between sampling stations for the eDNA (N = 27, red bold line) and TEF samples (N = 30, blue dashed line) for each 10-km distance class (76.5 to 493 km downstream from Lake Geneva). The filled circles (eDNA) and filled triangles (TEF) represent significant spatial correlations between the fish assemblages on the correlogram.

When considering the longitudinal profile of the river, Mantel tests between Bray-Curtis dissimilarity matrices and the longitudinal distance matrix were significant for both eDNA metabarcoding data (P < 0.01) and TEF data (P < 0.01). Mantel correlograms performed on the eDNA and TEF samples showed significant spatial autocorrelations across spatial extents of 70 km and 40 km, respectively (Fig. 7).

### eDNA metabarcoding detection distance

**Figure 8:**
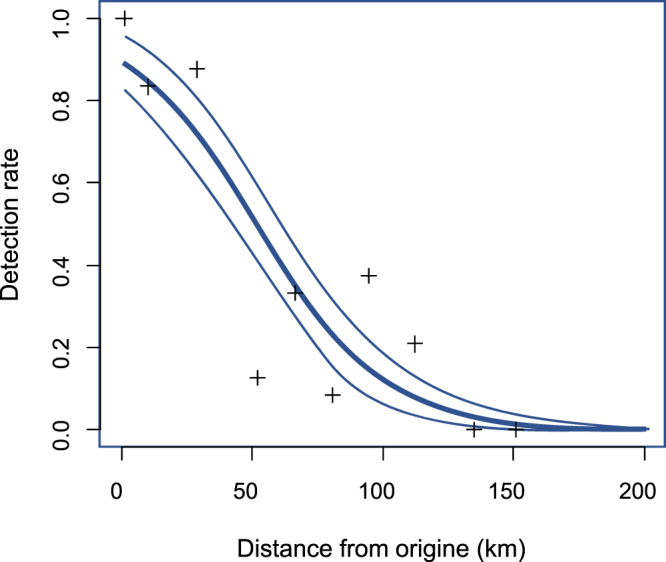
Observed and predicted detection rate of *Coregonus lavaretus* downstream from B-SE river section’s dam. Glm model predictions (blue thick line) and prediction area delimited by the standard error (blue fine lines). Detection rate was determined as the number of positive amplifications of *C*. *lavaretus* DNA in 24 PCR replicates.

Whitefish (*Coregonus lavaretus*) is a lake-dwelling species for which eDNA was detected in abundance up to 60 km downstream from Lake Geneva (river section B-SE, 8.9% of the total number of reads) and in the two tributaries connected to the Annecy and Le Bourget lakes (kilometer point 64 and KP 80). Whitefish had a positive detection for less than 9 PCR replicates when the distance from the DNA source (river section B-SE’s dam) was higher than 50 km and was no more detected at KP 195. The eDNA travelling time was estimated to be 41.7 hours for the first 100 km. The detection threshold predicted by the glm model (Fig. 8) fell below 4% at 130 km (±std error: 121–141 km).

According to V_dep_ values reported in the literature for fine particle organic matter (FPOM) (Supplementary Table 4), the median V_dep_ value was approximately 0.180 mm.s^−1^ (interquartile range 0.08–0.43). Using this median V_dep_ value, our simulations allowed correct predictions of the eDNA detection distance ranges observed in experimental and observational studies previously reported in the literature^22,26,29,30^ and in this study when the corresponding hydrological conditions were considered (Table 2).

**Table 2:** Comparison of eDNA detection distances observed in caged fish experiments (Exp) or observational studies (Obs) and those predicted by simulation considering a value of the vertical transfer of FPOM from the water column to the riverbed (V_dep_) computed from selected publications (median value ± interquartile range, see Supplementary Table 4) The maximal predicted detection distance is defined as the distance for which only one mtDNA copy of a hypothetical MOTU quantity released upstream is still present in the water column (see Method section for explanation).

Reference	Method	DNA analysis	Waterflow (m^3^.s^−1^)	Wetted width (m)	Observed distance of detection	Predicted distance of detection (±Interquartile Q1–Q3)
Jane *et al*. (2015)	Exp	qPCR	0.003	1.19	>239.5 m	128 m (54–283)
			0.031	3.35	>239.5 m	443 m (186–979)
Wilcox *et al*. (2016)	Exp	qPCR	0.006	1.19	41 to 222 m	235 m (98–518)
	Exp		0.010	3.35	72 to 1,459 m	137 m (57–303)
	Obs.		0.059	2.88	100 to 900 m	965 m (404–2131)
Civade et al. (2016)	Obs.	meta barcoding	0.170	1.80	1.7 to 3.6 km	4.5 km (1.9–9.9)
Deiner et Altermatt (2014)	Obs	qPCR	3.520	14.00	>9.1 km	11.9 km (5–26.3)
			3.520	14.00	>9.1 km	12.8 km (5.4–28.3)
			3.790	14.00	1.6 to 9.1 km	11.9 km (5–26.3)
This study	Obs	meta barcoding	436.00	132.00	130.0 km (120.7–141.1)	156.1 km (65.4–344.9)

The water velocity (u) and the water depth (h) used to compute Sp and the maximal detection distance are approximated from the mean water flow (Q in m^3^) and the mean wetted width (w) considering a channel with a rectangular cross section.

## Discussion

Comparing the eDNA metabarcoding sampling campaign and the TEF long-term surveys demonstrated the capacity of this approach to qualitatively and quantitatively (relative abundance) reveal the longitudinal patterns of fish biodiversity along a large river. Combining field data and a literature review, we also showed that eDNAm behaves in the river water column similarly to FPOM and that its detection distance is mainly dependent on the hydraulic characteristics of the river channel.

Our reference database Teleostei^21^ covers all the fish biodiversity in the Rhône catchment. However, some molecular marker did not discriminate between species (Supplementary Table 2). Diversity of some genera was reduced from 7 to 3 species (*Carassius*, *Leuciscus* and *Salvelinus*), and identifying new markers to overcome this limitation is important when markers detect phylogenetically distant species (Cypr_1 and Cypr_2).

Of the species that were detected by only eDNA and were previously unknown in the Rhône River, four (e.g., *S*. *salar*, *A*. *regius*, *D*. *labrax*) are consumed in large quantities, and their DNAs most likely originated from treatment plants from the cities (Geneva, Lyon). In addition to being marketed as food, *O*. *mykiss* and *Salvelinus spp*. are subject to fish farming^43^, and their DNAs are also released into the river with fish farm effluents. The abundance of *O*. *mykiss* eDNA could also have been related to the release of individuals by fishery associations at the time of our eDNA sampling campaign, a few weeks after the fishing season opening. Such eDNA transfer in rivers limits the investigation of commonly consumed or manipulated species^28^, and eDNA sampling too close to the outlet of a lake or confluence with a tributary should be avoided.

Excluding estuarine species, 41 of the 48 species occurring along the Rhône River^43^ were detected by eDNA metabarcoding, whereas 43 species were detected by the 10-year TEF surveys. Interest in using the eDNA method to monitor rare or elusive fish species has already been emphasized^45^. Two critically endangered species and difficult to capture by electrofishing (nocturnal benthic species), the Apron (*Z*. *asper*) and the weather loach (*M*. *fossilis*), were detected in the Rhône River directly downstream from respectively the confluence of the Ardèche River and backwaters (section F-SB) where they are known to be present^43^. eDNA method was also more efficient than TEF to differentiate the thicklip grey mullet (*C*. *labrosus*) and the flathead grey mullet (*M*. *cephalus*) from the thinlip grey mullet (*Liza ramada*), another estuarine species present in TEF samples.

Detection rates of a given MOTU vary with the amount of eDNA analyzed and the corresponding species abundance^46^ and are used to evaluate the frequency of a MOTU between sites^30^. The significant relationships between the standardized number of reads and the detection rates for each of the 29 MOTU/taxa demonstrated the validity of between sites comparison of the relative abundance of a given MOTU/taxa. Furthermore, our glm model including all the MOTUs highlighted also that the relative abundance of MOTUs are globally comparable between them and between sites. However, the significance of the MOTU’s identity effect showed that a given detection rate did not correspond to the same standardized number reads according to the considered MOTU. But the deviance explained by this fixed effect is limited and the discrepancies between species allow to distinguish between relative abundance classes, a sufficient level of precision for biomonitoring.

When comparing the eDNA metabarcoding and TEF methods on the local scale (few km), the eDNA samples produced a more comprehensive species list than the TEF samples. eDNA metabarcoding has already been recognized as a more sensitive method than electrofishing^22,29^ or netting^20,21,47,48^ for fish detection. The similarity between the faunistic list of an eDNA sample and the cumulative list of species caught during all TEF campaigns demonstrates that all species detected by eDNA could be locally present. The large homing range of most of fish species^49^ in combination with species rarity, can lead to very low detection probabilities with TEF. Most importantly, traditional methods are insufficient for providing a comprehensive species list with a limited sampling effort in large rivers^6,50^. When the sampling effort was higher and different fishing methods were used in addition to TEF, the number of species caught in one traditional sampling campaign was comparable to that detected by eDNA metabarcoding with our workflow (Danube River study, Supplementary Table 3).

In addition to comprehensively estimating species richness, we observed a significant correlation (of medium intensity) between species relative abundance (TEF) and the number of standardized reads. Similar results were previously obtained with the metabarcoding approach^12,16,20^, but contrasting results were also observed^19,39,51^. The differences between TEF and eDNA metabarcoding can be related to the fact that in rivers more than two meters deep, TEF is possible only along the bank. Comparisons with other traditional fishing methods (gill nets, longline) in large rivers^6,51^ confirm our results: *A*. *alburnus* and *S*. *cephalus*, two surface-oriented species, are overrepresented by TEF, whereas several species are underrepresented by TEF because they preferentially position themselves in the bottom layer of the water column (*B*. *barbus*, *A*. *brama* and *S*. *lucioperca*) or on the bottom (*G*. *cernua* and *B*. *barbatula*)^43^. eDNA gives probably a better integrated view of fish assemblages in large rivers than classical methods.

The Shannon index values computed from the eDNA samples were unsurprisingly higher than those obtained from the annual TEF samples because of the differences in species richness. However, the higher Evenness index values achieved with eDNA demonstrate a better repartition of individuals between species than that obtained from TEF. We hypothesize that due to the turbulent conditions prevailing in rivers, the homogenization of eDNA is easier and makes MOTU detection more stable between sampling occasions^15^.

When eDNA and TEF samples were pooled on a larger scale (14 river sections), the species relative abundances of the fish assemblages revealed by the two methods were highly correlated. The succession of the dominant species along the 20 successive river sections as presented by the eDNA samples (Supplementary Figure 2) agrees with previous descriptions^52^. Cool water species are located upstream (*Esox Lucius*, *Cottus sp*, *Leuciscus spp*) at the opposite of estuarine species (*L*. *ramada*) while *Rhodeus sericeus* is abundant in midstream river sections. eDNAm also highlights the upstream migration of twaite shad (*Alosa spp*) during the sampling period^43^ and the regular decrease in eel (*Anguilla Anguilla*) upstream, demonstrating the potential of eDNA metabarcoding to monitor anadromous species. Notably, such a comprehensive description of large-scale fish biodiversity patterns obtained in less than two weeks is not feasible with traditional fish sampling techniques.

The significant spatial autocorrelation between TEF samples up to 40 km shows the similarity of fish assemblages on the river section scale. The low diversity of aquatic habitats in such a heavily channelized and impounded river tends to decrease the spatial variability of fish assemblages^52^. However, the spatial autocorrelation between the eDNA samples was significant within 70 km, which indicates an influence of the upstream production of eDNA far downstream due to its transportation.

Studying the downstream transport of eDNA and the review of literature regarding FPOM transport in streams makes discussing this last question feasible. We used the detection rate decrease in whitefish downstream from the outlet of three alpine lakes to evaluate its detection distance, assuming a constant release of whitefish eDNA from these lakes. As we did not consider the potential degradation of eDNA during the two-day transport period^10,11^, we most likely underestimated the potential transport distance (S_p_). Conversely, the presence of occasional whitefish individuals within the Rhône River might have led to a slight overestimation of this distance. Our detection distance (130 km) is much higher than those already estimated experimentally in small streams (less than one km)^26,29^ but also from dwelling species at the outlet of lakes (1.7 km to more than 9.1 km)^22,30^.

Using an estimation of deposition velocity (V_dep_) based on previous studies (Supplementary Table 4) allowed to predict correctly the range of eDNA’s detection distance observed in previous works and in this study (Table 2). The hypothesis that the downstream decrease in eDNA is comparable to that observed for FPOM and highly dependent of the local hydraulic characteristics is confirmed. eDNA behaves like FPOM not only in small streams^29^ but also in large rivers. Deposition velocity in streams is a balance between upward turbulent mixing and gravitational settling^44^. V_dep_ hardly varies among particle types and size of FPOM but tend to increase significantly in streams with flows below 100 L.s^−1^ in relation to the proximity and roughness of the sediment (shear stress, transport storage zone)^53^. Unlike V_dep_, S_p_ is the product of both deposition and transport characteristics, and deep, fast rivers transport FPOM further than shallow rivers^44^. The scale at which eDNA reveals fish assemblage structures seems highly dependent of the river size. Simulations of the detection distance (Supplementary Figure 3) show that in small streams, eDNA transportation distance are comparable to the scale at which traditional sampling technics are performed (from less to few km). As the river size increases, eDNA is conveyed further downstream and deliver a more spatially integrated measure of biodiversity which is decoupled from a physical local habitat^31^. But, in a large catchment as the Rhône River, eDNA collected downstream does not detect anymore the fish species which are dominant upstream (Supplementary Figure 2). To our opinion, the statement that eDNA sampling allows “an estimate of catchment-level diversity, including both aquatic and terrestrial taxa”^31^ must be relativized according to the size of the watershed. In addition, as eDNA is subject to deposition velocity, the relative amount of eDNA originating from upstream decreases faster in comparison with the amount of eDNA released locally by fish species. Then, when analyzing not only species occurrence but also relative species abundance based on the standardized number of reads, our study demonstrates that eDNA reveals a correct picture of fish assemblage structures in a large river despite its downstream transportation. However other catchment systems of various size and with different species pools must be investigated to better understand the complex spatiotemporal dynamics of the information delivered by eDNA in rivers^33^.

To our knowledge, our results are the first demonstration of the capacity of eDNA metabarcoding to describe large-scale quantitative patterns of fish assemblages in rivers. Compared to the electrofishing technique, eDNA provides a comparable image of the fish assemblage but integrates a larger extent than the traditional sampling location both upstream and within the entire cross river section. Our eDNA workflow combined into one single sampling campaign of 12 days is as efficient as ten years of traditional sampling effort (approximately 300 days of field work) at obtaining an accurate image of fish biodiversity on the watershed scale. Making precise inferences on fish abundance or biomass from eDNA metabarcoding is unrealistic, and the quantity of eDNA released by the fish is most likely also related to specific metabolism rates^40^. However, eDNA metabarcoding can deliver valuable information about relative species abundance and be useful for monitoring purposes^48^. The large quantity of water sampled ensures the detection of most rare species with only two replicates, and *in situ* filtration prevents the risks of contamination and DNA degradation prior to analysis^21^. In practice, to limit the potential noise of transported eDNA and to ensure independence between eDNA samples in large rivers, the distance between the eDNA samples should be around 70 km. This distance depends on the river size, and a few kilometers between sampling sites should be sufficient in a smaller river. It is also recommended to avoid sampling close to locations where the introduction of exogenous fish eDNA to the river is suspected (e.g., tributaries, effluents). Indeed, despite the ecological and societal importance of large rivers, fish sampling in these systems remains costly, time consuming, and limited to specific habitats. eDNA metabarcoding thus appears to be a reliable, cost-effective method^45^ for screening quantitative fish biodiversity patterns on a large-scale.

## Methods

### Study area

From the France-Switzerland border (Rhône River kilometer point KP 24) to the Mediterranean Sea (KP 540), successive dams (Fig. 1) delimit 20 river sections^52^ (Supplementary Table 1). The two first sections (A-GE, B-SE) are deep impoundments directly connected to Lake Geneva (KP 0 to 60). Downstream, seventeen hydropower schemes separated by free-flowing sections (main channel MC) follow each other (Fig. 1), and 16 consist of a bypass reach (BPS, former riverbed) and a diversion canal (MC) with a hydropower plant. The final river section (T-PA), including the main channel and a secondary arm (DELT, Deltaïc Rhône), delimits the Island of Camargue (see Supplement Note 2 for the main hydromorphological and physico-chemical characteristics of the river).

### eDNA sampling, *in situ* filtration and treatment

A total of 59 water samples (Supplementary Table 1) were collected (KP 24.5 to KP 548) in each of the 20 successive sections of the Rhône River (MC, BPR and DELT: respectively 31, 20 and 8 samples) from April 6th to May 13^th^, 2016. The average discharge was 2154 m^3^.s^−1^ (1184 to 3115 m^3^.s^−1^). eDNA sampling was performed using a filtration device (VigiBOAT, SPYGEN, le Bourget du Lac, France) composed of a peristaltic pump (nominal flow of 1.1 L.min^−1^), a VigiDNA® 0.45-µM cross flow filtration capsule (SPYGEN, le Bourget du Lac, France) and disposable sterile tubing for each filtration capsule (Supplementary Figure 4). Two filtrations were performed in parallel at each site, each timed at 30 min for a water volume of approximately 30 L. A preliminary study on the influence of the sampling effort on eDNA detection showed that two filtrations were sufficient to detect more than 95% of the local species richness (Supplementary Figure 1). At the end of each filtration, the water inside the capsule was emptied, and the capsule was filled with 80 mL of CL1 Conservation buffer (SPYGEN, le Bourget du Lac, France) and stored at room temperature.

DNA extraction, amplification, high-throughput sequencing, sequence analyzing, and taxa assignment are described in Supplementary Note 1.

For convenience, all MOTUs are referred to as species in the text (Supplementary Table 2). Species not previously recorded in the Rhône River^43^ were not considered in the subsequent data analysis. Two implausible detections by eDNA of estuarine fish species downstream from Lake Geneva (*Atherina boyeri* and *Dicentrarchus labrax*) were also discarded.

As the total number of DNA copies varied between sites, we standardized the reads numbers to ensure that the numbers of reads per species were comparable between sites^32^ and that they can be interpreted in terms of relative abundance. All eDNA samples were resampled to randomly select 163,121 reads per site (R package MASS^59^, function sample without replacement), which was the smallest total number of reads found at one site. All species detected in the initial dataset were still found after resampling.

### Relation between detection rate and number of MOTU’s copies

We modelled the relationship between the standardized read number of each MOTU (log-transformed) and its detection rate (proportion of positive amplifications in 24 PCR replicates per site) among sites using generalized models (R software, package MASS^59^, function glm, binomial error). Model’s residual deviance was used as the goodness-of fit criterion in the model evaluation. We considered the 24 MOTUs covering most of the detection rate’s range (3 to 23 positive PCR) and present in more than 10 sites. We modelled separately the relationship for each of the 24 MOTUs. Then we performed the model on the complete dataset, adding MOTU’s identity and the interaction with the number of reads.

### Comparison of eDNA metabarcoding and TEF samples on the sampling site scale

Traditional electrofishing (TEF) was performed every year or two years along the entire Rhône River course (Fig. 1) for one decade (2006–2016) as part of regular surveys operated by Electricité de France (EDF, 20 sites) and the National French Agency for Biodiversity (AFB, 20 sites). TEF sampling methods are described in Supplementary Note 2.

Preliminary analysis showed that ten annual TEF samples were necessary to not underestimate the species richness (species accumulation curves, not presented here). Thus, only the TEF sites sampled every year (n = 25) were considered to compare TEF and eDNA samples. For all TEF samples we performed generalized linear model to ensure that the species richness per site did not show any consistent trend during the studied decade and pooled all the sampling sessions per site.

On the local scale (sampling location), the pairs of TEF and eDNA samples to be compared were required to be from the same type of river reach (MC, BPS or DELT). When different pairs of TEF and eDNAm sites could be selected from the same river section, the least distant pair of sites was selected. Finally, a total of 16 river locations (L01 to L16) distributed along the entire river were retained (Supplementary Table 1). The distances between the eDNA samples and TEF occasions ranged from 0.2 to 7 km (average of 3.6 km), a distance comparable to the recommended fishing length for large rivers (20 times the river width, i.e.2.8 to 8.8 km for our sites)^55^. The species richness, Shannon index and Evenness index were computed using the package vegan (R software, functions specnumber and diversity)^54^.

### Longitudinal patterns of fish biodiversity revealed by eDNA metabarcoding and TEF

To compare quantitatively (relative abundance) the fish assemblages described by metabarcoding and TEF methods on a large scale (from Lake Geneva to the sea), the eDNA and TEF samples were pooled within each of the successive river sections where the two methods were available (14 of the 20 river sections) and log-transformed.

The longitudinal patterns of fish assemblages revealed by eDNA and TEF were compared using co-inertia analysis (R software, package ade-4^57^, functions dudi.pca and coinertia). This multivariate method allows the comparison of the ordinations of two datasets to find the orthogonal co-inertia principal components (PC) that maximize the co-inertia (or co-variance) between the two datasets^57^. This method is especially suitable when many species are sampled from a few sites. First, we conducted separate analyses of the TEF and eDNA datasets with two centered principal component analyses (PCAs) and then processed the co-inertia analysis on the principal components of the first two PCAs of each dataset. The RV co-inertia criterion (0 to 1) measures the adequacy between the two tables^57,58^ and its significance (Monte-Carlo test, 10,000 permutations).

To comprehensively describe the quantitative patterns of fish biodiversity along the Rhône River as revealed by eDNA, the eDNA samples were pooled in each of the 20 river sections and analyzed with a centered PCA.

For each sampling method, we performed a Mantel test (R software, package vegan^56^, functions mantel and mantel.randtest) to evaluate whether the similarity between samples located on the main river course (TEF: 31 sites, eDNA: 39 sites) was dependent on the distance between the sampling sites. We used the Bray-Curtis index as a measure of the dissimilarity between assemblages and performed 999 permutations for 19 geographic distance classes (ten-km width) among the sites. For each sampling method, a correlogram (R software, package vegan^56^, function mantel.correlog) was performed to determine the maximal geographic distance between samples for which the spatial autocorrelation remained significant.

### eDNA metabarcoding detection distance

Whitefish (*Coregonus lavaretus*) is abundant in the three Alpine lakes connected to the upper Rhône^43^ but is only found occasionally in the Rhône River itself^52^ downstream from section B-SE: only one individual reported in our TEF dataset, and less than one-thousandth of the total catch by fishermen^59^. We used generalized linear model (R package MASS, function glm) to analyze detection rate of eDNA relative to the downstream distance of the sampling site from section B-SE. The maximal detection distance was defined as distance before detection drops below a 4% threshold (less than 1 PCR positive on 24)^30^. We tested the hypothesis that the downstream decrease in eDNA was mainly due to a sedimentation rate similar to that observed for fine particulate organic matter (FPOM)^29,44^.

The vertical transfer of FPOM from the water column to the riverbed (V_dep,_ deposition velocity) was expressed as V_dep_ = u.h/S_p_ (Eq. 1), with S_p_ the distance in m needed to retain 63.2% of the FPOM in the riverbed, u the mean water velocity (in m.s^−1^) and h the mean water depth (in m)^44^. According to the V_dep_ values reported in the literature (Supplementary Table 4) we compared the eDNA’s detection distance observed in experiments or observational studies (including this study) and the eDNA’s detection distance predicted by V_dep_ for the hydrological conditions prevailing in each previous study. The predicted maximal detection distance was defined as the distance for which only one mtDNA (mitochondrial DNA) copy of a MOTU released upstream was still present in the water column, considering an initial released quantity of 2000 mtDNA copies/L multiplied by 2.5, to simulate taking a 2.5-L sample^29^. All statistical analyses were done with the program R, version 3.3.3^60^.

### Data Availability

All Illumina raw sequences data are available on Dryad 10.5061/dryad.t4n42rr.

## Electronic supplementary material

Supplementary Material
