# Supplementary material for: Environmental DNA reveals quantitative patterns of fish biodiversity in large rivers despite its downstream transportation
Source: Sci Rep. 2018 Jul 10;8:10361. doi: 10.1038/s41598-018-28424-8 (PMC6039509; doi:10.1038/s41598-018-28424-8)

Environmental DNA reveals quantitative patterns of fish biodiversity in large rivers despite its downstream transportation

Supplementary Tables and Figures

Didier Pont [1, 2, 3 *], Mathieu Rocle [4], Alice Valentini [1, 2], Raphaël Civade [2], Pauline Jean [1], Anthony Maire [5], Nicolas Roset [6], Michael Schabuss [7], Horst Zornig [7] & Tony Dejean [1, 2]

1 SPYGEN, 17 rue du Lac Saint-André, Savoie Technolac, 73370 Le Bourget du Lac, France. 2 VIGILIFE, 17 rue du Lac Saint-André, Savoie Technolac, 73370 Le Bourget du Lac, France. 3 Institute of Hydrobiology and Aquatic Ecosystem Management (IHG), University of Natural Resources and Life Sciences, Gregor-Mendel-Strasse 33, 1180 Vienna, Austria. 4 Compagnie Nationale du Rhône, Direction de l'Ingénierie, 2 rue André Bonin, 69004 Lyon. 5 EDF R&D, LNHE (Laboratoire National d'Hydraulique et Environnement), 6 quai Watier, 78401 Chatou Cedex, France. 6 French National Agency for Biodiversity, Auvergne-Rhone-Alpes Regional Directorate, Parc de Parilly, Chemin des Chasseurs, 69500 Bron. 7 PRO FISCH OG Ecological Consultants, Hörlgasse 6, A-1090 Vienna, Austria. Correspondence and requests for materials should be addressed to D.P. (email: didier.pont@boku.ac.at)

List of supplementary Notes Tables and Figures

Suppl. Note 1: DNA extraction, amplification, high-throughput sequencing, sequence analysing and taxa assignement

Suppl. Note 2: Traditional Electrofishing Methods

Suppl. Table 1: eDNA and TEF sampling site characteristics

Suppl. Table 2: List of species and MOTUs recorded in the Rhône River

Suppl. Table 3: Comparison of eDNA and traditional sampling methods on the Danube River

Suppl. Table 4: Literature review of depositional velocity and transport distance of fine particulate organic matter (FPOM) in stream and river

Suppl. Fig.1: Influence of sampling effort on eDNA metabarcoding detection efficiency

Suppl. Fig. 2: Longitudinal profile of species abundances along the main channel of the Rhône River as revealed by eDNA metabarcoding and traditional electrofishing sampling

Suppl. Fig. 3: Simulated maximal detection distance of eDNA

Suppl. Fig. 4: Water filtration device sampler and cross-flow filtration capsule used to sample eDNA

Supplementary Note 1: DNA extraction, amplification, high-throughput sequencing, sequence analyzing and taxa assignment

DNA extraction, amplification, high-throughput sequencing

DNA extraction was performed in a dedicated room for water DNA sample extraction, equipped with positive air pressure, UV treatment and frequent air renewal. Before entering this extraction room, personnel changed into full protective clothing comprising disposable body suit with hood, mask, laboratory shoes, overshoes and gloves in a connecting zone. All benches were decontaminated with 10% commercial bleach before and after each manipulation. For DNA extraction, each filtration capsule, containing the CL1 buffer, was agitated for 15 min on an S50 shaker (cat Ingenieurbüro™) at 800 rpm and then the buffer was emptied into a 50-mL tube before being centrifuged for 15 min at 15,000×g. The supernatant was removed with a sterile pipette, leaving 15 mL of liquid at the bottom of the tube. Subsequently, 33 mL of ethanol and 1.5 mL of 3M sodium acetate were added to each 50-mL tube and stored for at least one night at -20°C. The tubes were centrifuged at 15,000 × g for 15 min at 6°C, and the supernatants were discarded. After this step, 720 µL of ATL buffer from the DNeasy Blood & Tissue Extraction Kit (Qiagen) was added. The tubes were then vortexed, and the supernatants were transferred to 2-mL tubes containing 20 µL of Proteinase K. The tubes were finally incubated at 56°C for two hours. Subsequently, DNA extraction was performed using NucleoSpin® Soil (MACHEREY-NAGEL GmbH & Co., Düren Germany) starting from step 6 and following the manufacturer's instructions. The elution was performed by adding 100 µL of SE buffer twice. After the DNA extraction the samples were tested for inhibition by qPCR (Biggs et al. 2015). If the sample was considered inhibited it was diluted 5-fold before the amplification.

DNA amplifications were performed in a final volume of 25 µL, using 3 µL of DNA extract as the template. The amplification mixture contained 1 U of AmpliTaq Gold DNA Polymerase (Applied Biosystems, Foster City, CA), 10 mM Tris-HCl, 50 mM KCl, 2.5 mM $MgCl_2$, 0.2 mM each dNTP, 0.2 µM "teleo" primers (Valentini et al. 2016), 4 µM human blocking primer for the "teleo" primers (Civade

et al. 2016) and 0.2 µg/µL bovine serum albumin (BSA, Roche Diagnostic, Basel, Switzerland). The "teleo" primers were 5'-labeled with an eight-nucleotide tag unique to each PCR replicate (with at least three differences between any pair of tags), allowing the assignment of each sequence to the corresponding sample during sequence analysis. The tags for the forward and reverse primers were identical for each PCR replicate. The PCR mixture was denatured at 95°C for 10 min, followed by 50 cycles of 30 s at 95°C, 30 s at 55°C and 1 min at 72 °C and a final elongation step at 72°C for 7 min in a room dedicated to amplified DNA with negative air pressure and physical separation from the DNA extraction rooms (with positive air pressure). Twelve replicate PCRs were run per filtration, i.e., 24 per sampling site.

After amplification, the samples were titrated using capillary electrophoresis (QIAxcel; Qiagen GmbH) and purified using the MinElute PCR purification kit (Qiagen GmbH). Before sequencing, purified DNA was titrated again using capillary electrophoresis. The purified PCR products were pooled in equal volumes to achieve a theoretical sequencing depth of 500,000 reads per sample. PCR purification were performed in a room dedicated to amplified DNA analysis with negative air pressure and physically separated from the eDNA extraction room. All benches were decontaminated with 10% commercial bleach before and after each manipulation. Library preparation and sequencing were performed at Fasteris (Geneva, Switzerland). The libraries were prepared using the MetaFast protocol (Fateris, https://www.fasteris.com/dna/?q=content/metafast-protocol-amplicon-metagenomic-analysis), and paired-end sequencing (2x125 bp) was carried out on an Illumina HiSeq 2500 sequencer (Illumina, San Diego, CA, USA) with the HiSeq SBS Kit v4 (Illumina, San Diego, CA, USA) following the manufacturer's instructions. In total, eleven libraries were sequenced on a single HiSeq run. Nine negative extraction controls and ten negative PCR controls (ultrapure water, 12 replicates) were amplified and sequenced in parallel to the samples to monitor possible contaminants.

Sequence analysis and taxa assignment

Sequence reads were analyzed using programs implemented in the OBITools package (http://metabarcoding.org/obitools) (Boyer et al. 2016) following a protocol already described (Valentini et al. 2016). The forward and reverse reads were assembled using the *illuminapairedend* program using a minimum score of 40 and retrieving only joined sequence. The reads were then assigned to each sample using the *ngsfilter* program.

A separate data set was created for each sample by splitting the original data set in several files using *obisplit*. After this step, each sample was analyzed individually before merging the taxon list for the final ecological analysis. Strictly identical sequences were clustered together using *obiuniq*. Sequences shorter than 20 bp, or with occurrence lower than 10 were excluded using the *obigrep* program. The *obiclean* program was then run within a PCR product. All sequences labelled 'internal' that correspond most likely to PCR substitutions and indel errors were discarded. Taxonomic assignment of the MOTUs was performed using the program *ecotag* with the local reference database Teleostei (Valentini et al. 2016). MOTUs showing less than 98% similarity to the local reference database were removed. Finally, considering the bad assignments of a few sequences to the wrong sample due to tag-jumps (Schnell et al. 2015), all sequences with a frequency of occurrence below 0.001 per taxon and per library were discarded. These thresholds were empirically determined to clear all reads from the negative controls included in our global data production procedure (De Barba et al. 2014).

For each site, the results from the two filtrations and the 24 PCRs were summed to obtain the total number of reads per taxa and per site. In total, 57,290,839 reads (44% of the initial number of reads before filtering) were assigned to 48 taxa. Taxa present in only one PCR replicate and only one sample replicate were discarded (Ficetola et al. 2015). The molecular markers used for fish detection did not discriminate between species belonging to different genera for two groups. They are referred to as Cypr_1 (*Telestes souffia*, *Chondrostoma nasus* and *Parachondrostoma toxostoma*) and Cypr_2 (*Hypophthalmichthys molitrix* and *Ctenopharyngodon idella*). Within some genera, the following

species were not differentiated: *Salvelinus alpinus* and *S. fontinalis* (Sal_spp); *Leuciscus idus* and *Leuciscus* (Leu_spp); *Carassius*, *Carassius auratus* and *Carassius gibelio* (Cas_spp); *Alosa fallax* and *Alosa* (Alo_spp); *Cottus gobio* and *Cottus petiti* (Cot_sp); and *Lampetra planeri* and *Lampetra fluviatilis* (Lam_spp).

References:

Biggs, J., Ewald, N., Valentini, A., Gaboriaud, C., Dejean, T., Griffiths, R. A., Foster, J., Wilkinson, J. W., Arnell, A., Brotherton, P., Williams, P., Dunn, F. Using eDNA to develop a national citizen science-based monitoring program for the great crested newt (*Triturus cristatus*). *Biol. Conserv.* 183, 19-28 (2015).

Boyer, F., Mercier, C., Bonin, A., Le Bras, Y., Taberlet, P., & Coissac, Obitools: A unix-inspired software package for DNA metabarcoding. *Mol. Ecol. Resour.* 16, 176–182 (2016).

Civade, R., Dejean, T., Valentini, A., Roset, N., Raymond, J-C., Bonin, A., Taberlet, P. & Pont, D. Spatial representativeness of environmental DNA metabarcoding signal for fish biodiversity assessment in a natural freshwater system. *PLoS ONE* 11(6), 0157366; 10.1371/journal.*pone.0157366* (2016).

De Barba, M., Miquel, C., Boyer, F., Mercier, C., Rioux, D., Coissac, E., & Taberlet, P. DNA metabarcoding multiplexing and validation of data accuracy for diet assessment: Application to omnivorous diet. *Mol. Ecol. Resour.* 14, 306–323 (2014).

Ficetola, G. F., Pansu, J., Bonin, A., Coissac, E., Giguet-Covex, C., De Barba, M., Gielly, L., Lopes, C. M., Boyer, F., Pompanon, F., Rayé, G. & Taberlet, P. Replication levels, false presences and the estimation of the presence/absence from eDNA metabarcoding data. *Mol. Ecol. Resour.* 15, 543-556 (2015).

Schnell, I. B., Sollmann, R., Calvignac-Spencer, S., Siddall, M. E., Douglas, W. Y., Wilting, A. & Gilbert, M. T. P. eDNA from terrestrial haematophagous leeches as a wildlife surveying and monitoring tool–prospects, pitfalls and avenues to be developed. *Front. Zool.* 12, 1 (2015).

Valentini, A., Taberlet, P., Miaud, C., Civade, R., Herder, J., Thomsen, P. F., Bellemain, E., Besnard, A., Coissac, E., Boyer, F., Gaboriaud, C., Jean, P., Poulet, N., Roset, N., Copp, G. H., Genie, P., Pont, D., Argillier, C., Baudoin, J. M, Peroux, T., Crivelli, A. J., Olivier, A., Acqueberge, M., Le Brun, M., Møller, P. R., Willerslev, E. & Dejean, T. Next-generation monitoring of aquatic biodiversity using environmental DNA metabarcoding. *Mol. Ecol.* 25(4): 929-942 (2016).

Supplementary Note 2: Traditional electrofishing survey methods

Between Lake Geneva (Rhône River kilometer point zero, KP 0) and the Mediterranean Sea, the Rhône River is 540 km long, and its mean annual discharge gradually increases from 336 to 1700 $m3.s^{-1}$ (Olivier et al. 2009) due to the inputs of tributaries, with the Ain, Saône, Isère, Ardèche and Durance rivers being the most important. The slope of the Rhône watercourse is above 1‰ on the first hundred kilometers and varies between 0.26 and 0.76‰ thereafter. Even at 100 kilometers upstream of the river mouth, the slope remains high (0.66‰) and only decreases in the deltaic section (0.01‰). The river width increases from approximately 100 m to more than 700 m close to the sea. During average flow, the mean water velocity in the main channel (KP 61 to KP 476) is approximately 60 $cm.s^{-1}$ (standard deviation, std = 14 $cm.s^{-1}$), but higher mean water velocities are found upstream and downstream of the hydroelectric plants (80 $cm.s^{-1}$ (std = 12 $cm.s^{-1}$) and 120 $cm.s^{-1}$ (std = 34 $cm.s^{-1}$), respectively) (personal communication "Compagnie Nationale du Rhône"). The pH is always close to 8.0, and the mean annual water temperature ranges from 11.4 to 14.4°C along the river (Olivier et al. 2009).

Fish were sampled from a boat along the banks with only one pass, as recommended for large and non-wadable rivers (Fame Consortium 2005). Depending on the operator, fish were sampled either by point abundance sampling (Persat et Coop 1990), 30-minutes continuous sampling (Daufresne et al. 2015) [62] or systematic point sampling (Tomanova et al. 2013). EDF surveys were conducted two to four times a year, whereas the AFB surveys were conducted only once every year or two years but with a higher sampling effort (mean fishing time values of 23 min and 75 min, respectively). For the EDF surveys, we performed generalized linear model to ensure that the species richness per site did not show any consistent seasonal trend and pooled sampling sessions per year. To compare fish assemblages between sites, all fish species abundance was expressed as the equivalent of 162 min of effective sampling considering an equivalence of 18-min effective continuous sampling for 20-point sampling units (Daufresne et al. 2005, Pont et al. 1992). Finally, the EDF and AFB surveys included

214 and 141 samples, respectively (MC: 271, DELT: 14, BPS: 70), distributed among 14 of the 20 river sections.

References:

Daufresne, M., Veslot, J., Capra, H., Carrel, G., Poirel, A., Olivier, J-M. & Lamouroux, N. Fish community dynamics (1985–2010) in multiple reaches of a large river subjected to flow restoration and other environmental changes. *Freshw. Biol.* 60 (6), 1176–1191 (2015).

Fame Consortium *Manual for the application of the European Fish Index - EFI. A fish-based method to assess the ecological status of European rivers in support of the Water Framework Directive. Version 1.1* (Vienna, BOKU University, 2005).

Olivier, J-M., Carrel, G., Lamouroux, N., Dole-Olivier, M-J, Malard, F., Bravard, J.P. & Amoros, C. *The Rhône River Basin. In Rivers of Europe* (eds Tockner, K. Uehlinger, U. & Robinson, C.T.) (Elsevier, Amsterdam, 2009).

Persat, H. & Coop, G. H. *Electric fishing and point abundance sampling for the ichthyology of large rivers*. In Developments in electric fishing (ed.Cowx, I.G.) (Fishing News Books Ltd., Blackwell Scientific Publications, Oxford, 203-215, 1990).

Pont, D., Poizat, G. & Torre, F. *Etablissement d'une méthodologie pour un suivi piscicole du Rhône. Schéma de Vocation Piscicole du Rhône*. Rapport final à Délégation de bassin RMC et Agence de l'eau RMC. (1992).

Tomanova, S., Tedesco, P. A., Roset, N., Berrebi Di Thomas, R. & Belliard, J. 2013. Systematic point sampling of fish communities in medium- and large-sized rivers: sampling procedure and effort. *Fish. Manag. Ecol.* 20, 533–543 (2013).

Supplementary Table 1. eDNA and TEF sampling site characteristics.

Spatial coordinates, kilometric points (KP), river section codes, reach types (MC: main channel, BPS: by-pass section, DELT, Deltaïc Rhône) and sampling dates (April 6th to May 13th, 2016) of the eDNA and TEF samples. The numbers of years of the TEF survey are also specified. Depending on the local situation, eDNA samples were collected from a boat (59%), a bridge (38%) or the river bank (3%). The sampling distance between eDNA samples varied between 6.2 and 37.6 km (mean of 14.6 km). All the pairs of eDNA and TEF samples selected for local comparison between the two methods on the local scale are indicated (sampling locations: L01 to L16).

Names of the 20 river sections (11 to 48 km long, mean length of 25 km) delimited by the successive hydropower plants from upstream to downstream: A-GE: Génissiat, B-SE: Seyssel, C-CH: Chautagne, D-BE: Belley, E-BR: Brégnier-Cordon, F-SB: Sault-Brenaz, G-MI: Miribel-Jonage, H-PB: Pierre-Bénite, I-VG: Vaugris, J-PR: Péage de Roussillon, K-SV: Saint-Vallier, L-BV: Bourg les Valence, M-BE: Beauchastel, N-BL: Baix Logis Neuf, O-MO: Montélimar, P-DM: Donzère-Montdragon, Q-CA: Caderousse, R-AV: Avignon, S-VA: Vallabrègues, and T-PA: Palier-d'Arles (See locations in Fig. 1).

Geographical location				eDNAm samples		TEF survey	Sampling location
Longitude / Latitude	KP	River section	Reach type	Sampling collection	Sampling date	Nb. of Years	
5°57'50.0461" E / 46°8'39.2489" N	24.4	A-GE	MC	bridge	06/04/2016		
5°54'15.3176" E / 46°7'13.0998" N	31.1	A-GE	MC	bridge	06/04/2016		
5°48'37.7417" E / 46°4'25.7146" N	46.85	A-GE	MC	boat	06/04/2016		
5°48'55.9933" E / 45°59'3.3266" N	58.1	B-SE	MC	boat	06/04/2016		
5°48'49.6868" E / 45°53'5.3977" N	70.1	C-CH	MC	boat	07/04/2016		

Geographical location				eDNAm samples		TEF survey	Sampling location
Longitude / Latitude	KP	River section	Reach type	Sampling collection	Sampling date	Years	
5°49'12.0529" E / 45°52'13.2582" N	72.95	D-BE	BPS	boat	07/04/2016		
5°48'19.3806" E / 45°51'11.6482" N	74.6	D-BE	BPS			5	
5°47'50.7808" E / 45°49'52.5821" N	76.5	D-BE	MC			6	
5°47'1.3279" E / 45°45'4.9208" N	85.6	D-BE	BPS			7	
5°42'27.0745" E / 45°45'15.8710" N	89.1	D-BE	MC	bridge	07/04/2016		
5°45'3.1248" E / 45°42'22.1198" N	92.1	E-BR	BPS	bridge	07/04/2016		
5°43'34.6012" E / 45°42'31.8586" N	94.95	E-BR	BPS	boat	07/04/2016		
5°39'42.0602" E / 45°38'18.2792" N	107.3	E-BR	MC			5	
5°36'43.7713" E / 45°38'28.1832" N	112	E-BR	MC	boat	14/04/2016		
5°36'55.6996" E / 45°37'28.8332" N	113.5	F-SB	BPS	bridge	25/04/2016		
5°36'24.5153" E / 45°38'21.4699" N	115.5	F-SB	BPS			5	
5°35'19.7167" E / 45°39'20.7493" N	118	F-SB	BPS	boat	14/04/2016		
5°32'53.6338" E / 45°41'56.7114" N	124.3	F-SB	MC			10	L01
5°32'54.7976" E / 45°42'45.6948" N	126.3	F-SB	MC	bridge	25/04/2016		L01
5°26'2.0389" E / 45°47'57.7205" N	140.8	F-SB	MC	boat	14/04/2016		
5°24'29.4520" E / 45°51'15.6578" N	148.8	G-MI	BPS	bridge	25/04/2016		
5°20'50.6123" E / 45°52'57.9936" N	154.7	G-MI	MC	bridge	25/04/2016		L02
5°20'53.0192" E / 45°52'57.5900" N	154.7	G-MI	MC			5	
5°17'56.2812" E / 45°50'17.2464" N	160.5	G-MI	MC			11	L02

Geographical location				eDNAm samples		TEF survey	Sampling location
Longitude / Latitude	KP	River section	Reach type	Sampling collection	Sampling date	Years	
5°17'37.4316" E / 45°49'47.9550" N	162.2	G-MI	MC			11	
5°17'45.7897" E / 45°49'49.2445" N	162.2	G-MI	MC			11	
5°16'5.5571" E / 45°47'28.6706" N	167.3	G-MI	MC			11	
5°13'7.2181" E / 45°46'10.2972" N	171.4	G-MI	MC			11	
5°13'9.5171" E / 45°46'5.2252" N	171.4	G-MI	MC			11	
5°12'19.7075" E / 45°46'18.9818" N	172.3	G-MI	MC	bridge	25/04/2016		L03
5°12'2.5045" E / 45°46'20.1911" N	173.1	G-MI	MC			11	L03
5°10'3.5969" E / 45°47'44.1841" N	177.6	G-MI	MC			5	
5°5'27.6158" E / 45°48'42.1344" N	184.1	G-MI	MC			10	
4°56'51.0515" E / 45°49'10.4815" N	195.8	H-PB	BPS	bridge	25/04/2016		
4°49'45.8216" E / 45°44'43.0782" N	209.9	H-PB	MC	boat	14/04/2016		
4°49'19.4686" E / 45°38'52.1322" N	221.7	I-VG	BPS	boat	15/04/2016		L04
4°48'18.6815" E / 45°36'43.3148" N	225.8	I-VG	MC	boat	15/04/2016		
4°48'4.8766" E / 45°36'37.2996" N	226.1	I-VG	BPS			11	L04
4°48'48.3916" E / 45°34'8.7427" N	232.8	I-VG	MC			10	L05
4°50'29.5800" E / 45°32'33.5742" N	237.1	I-VG	MC	boat	15/04/2016		L05
4°46'5.7562" E / 45°27'25.6594" N	252.3	J-PR	MC	boat	15/04/2016		L06
4°44'37.7308" E / 45°24'47.4296" N	258.4	J-PR	MC			11	L06
4°45'20.6539" E / 45°23'55.4968" N	260	J-PR	MC			11	

Geographical location				eDNAm samples		TEF survey	Sampling location
Longitude / Latitude	KP	River section	Reach type	Sampling collection	Sampling date	Years	
4°46'5.1046" E / 45°21'27.5263" N	265.2	K-SV	BPS			11	L07
4°45'37.9595" E / 45°20'25.4299" N	267.4	K-SV	BPS	boat	03/05/2016		L07
4°47'23.4092" E / 45°18'58.3150" N	269.8	J-PR	MC	bridge	03/05/2016		L08
4°46'17.7679" E / 45°18'55.8569" N	270.6	K-SV	BPS			10	L09
4°47'42.1332" E / 45°18'10.1664" N	273.3	K-SV	BPS	boat	03/05/2016		L09
4°48'32.5548" E / 45°17'34.1473" N	274.1	K-SV	MC			11	L08
4°48'4.4006" E / 45°14'31.7537" N	279.9	K-SV	MC	bridge	03/05/2016		
4°48'43.9988" E / 45°11'2.9537" N	286.7	K-SV	MC			5	
4°48'46.5239" E / 45°7'8.0933" N	296	K-SV	MC	boat	03/05/2016		
4°49'6.9960" E / 45°6'25.1158" N	297.5	L-BV	BPS	boat	04/05/2016		
4°50'6.6138" E / 45°4'6.1511" N	302.1	L-BV	MC	bridge	03/05/2016		
4°51'12.1414" E / 45°0'52.0020" N	311	L-BV	MC	boat	04/05/2016		
4°51'30.2717" E / 44°58'7.6800" N	317.5	M-BE	BPS	boat	09/05/2016		
4°50'22.1755" E / 44°51'13.5742" N	330.6	M-BE	MC			5	
4°49'8.5861" E / 44°50'22.5726" N	333.4	M-BE	MC	boat	09/05/2016		
4°49'10.8390" E / 44°48'42.5884" N	336.3	N-BL	BPS	boat	09/05/2016		
4°45'18.6073" E / 44°45'51.7702" N	344.1	N-BL	MC	boat	09/05/2016		
4°47'12.5657" E / 44°41'10.8654" N	352.1	N-BL	MC	boat	09/05/2016		L010
4°46'24.2962" E / 44°41'55.3006" N	352.1	O-MO	BPS	boat	10/05/2016		

Geographical location				eDNAm samples		TEF survey	Sampling location
Longitude / Latitude	KP	River section	Reach type	Sampling collection	Sampling date	Years	
4°46'56.3210" E / 44°39'30.8614" N	355.6	O-MO	MC			11	L010
4°44'27.3757" E / 44°36'28.1624" N	361.6	O-MO	MC			11	L011
4°42'5.4889" E / 44°34'2.5673" N	367.3	P-DM	BPS			5	
4°43'34.1436" E / 44°32'4.5056" N	370.6	O-MO	MC	boat	10/05/2016		L011
4°41'26.5027" E / 44°30'48.4128" N	375	P-DM	BPS	boat	10/05/2016		
4°41'56.3986" E / 44°29'14.6846" N	376.8	P-DM	MC	boat	10/05/2016		L012
4°41'55.6530" E / 44°28'55.5020" N	377.6	P-DM	MC			11	L012
4°41'53.5052" E / 44°27'7.0384" N	380.7	P-DM	MC			8	
4°38'54.4873" E / 44°22'5.6532" N	391.1	Q-CA	BPS	bridge	11/05/2016		
4°38'55.7873" E / 44°22'1.7180" N	391.3	Q-CA	BPS			5	
4°43'58.6265" E / 44°21'4.3906" N	394.1	P-DM	MC			11	
4°44'11.0011" E / 44°19'41.7684" N	395.6	P-DM	MC			11	
4°44'15.6376" E / 44°19'19.2007" N	396.1	P-DM	MC			11	L013
4°44'12.3904" E / 44°19'22.7935" N	396.3	P-DM	MC	bridge	11/05/2016		L013
4°39'28.8680" E / 44°15'6.8296" N	404.1	Q-CA	BPS			11	L015
4°41'50.0687" E / 44°13'33.5161" N	410.2	Q-CA	MC	boat	10/05/2016		L014
4°40'24.1460" E / 44°12'57.5579" N	410.3	Q-CA	BPS	boat	10/05/2016		L015
4°42'38.1668" E / 44°12'37.0714" N	413.1	Q-CA	MC			11	L014
4°43'4.9634" E / 44°9'33.9037" N	418.8	Q-CA	MC	boat	10/05/2016		

Geographical location				eDNAm samples		TEF survey	Sampling location
Longitude / Latitude	KP	River section	Reach type	Sampling collection	Sampling date	Years	
4°43'18.3378" E / 44°4'48.4122" N	428.5	R-AV	BPS	boat	11/05/2016		
4°46'8.0785" E / 44°4'18.5146" N	433.1	R-AV	MC			10	L016
4°47'27.5615" E / 44°3'6.0512" N	436.8	R-AV	MC	boat	11/05/2016		L016
4°47'57.3619" E / 43°57'5.2744" N	453.3	S-VA	BPS	bridge	11/05/2016		
4°47'38.0436" E / 43°57'11.8084" N	453.4	S-VA	MC	bridge	11/05/2016		
4°42'2.0725" E / 43°53'56.5429" N	463.4	S-VA	MC	bridge	12/05/2016		
4°42'5.7564" E / 43°53'51.9428" N	463.5	S-VA	MC			5	
4°39'0.9799" E / 43°48'16.0456" N	478.5	T-PA	DELT	bridge	12/05/2016		
4°39'13.8996" E / 43°48'16.2184" N	478.5	T-PA	BPS	bridge	12/05/2016		
4°37'22.4962" E / 43°40'44.0821" N	493.6	T-PA	DELT			9	
4°36'17.8870" E / 43°40'5.1449" N	495.3	T-PA	DELT	boat	13/05/2016		
4°40'1.5924" E / 43°35'11.3863" N	507	T-PA	DELT	boat	13/05/2016		
4°27'9.2941" E / 43°39'56.8822" N	508.3	T-PA	DELT	bridge	12/05/2016		
4°27'10.7518" E / 43°39'57.3613" N	508.3	T-PA	DELT			5	
4°44'49.4718" E / 43°25'11.4877" N	527.9	T-PA	DELT	bridge	13/05/2016		
4°20'58.3872" E / 43°32'47.6117" N	533.8	T-PA	DELT	bridge	12/05/2016		
4°48'26.1724" E / 43°22'58.0127" N	534.2	T-PA	DELT	river bank	13/05/2016		
4°23'39.6884" E / 43°28'15.5856" N	547.8	T-PA	DELT	river bank	12/05/2016		

Supplementary Table 2. List of species and MOTUs recorded in the Rhône River

List of species and MOTUs recorded in the Rhône River downstream from Lake Geneva. The species list and taxonomic nomenclature follow that established by Keith et al. (2011). The list of estuarine species is not complete. When the molecular marker does not discriminate between several species, the abbreviation name corresponds to the grouping of these species.

Species status: D: diadromous species, E: estuarine species, F: freshwater species, M: marine species.

Species occurrences in the Rhône River (1), species occurrences only within the Rhone River basin (2) and species unknown in the Rhône River basin (3).

*: species detected but below the significance threshold

Species name	Common name	Species status	Species occurrence	Species occurrence in the TEF dataset	Detected MOTU's abbreviation name
Abramis brama	Common bream	F	1	+	Abr_bra
Alburnoides bipunctatus	Schneider	F	1	+	Alb_bip
Alburnus alburnus	Bleak	F	1	+	Alb_alb
Alosa fallax	Twaite shad	D	1	+	Alo_spp
Ameiurus melas	Black bullhead	F	1	+	Ame_mel
Anguilla anguilla	European eel	D	1	+	Ang_ang
Argyrosomus regius	Meagre	M	3		Arg_reg
Atherina boyeri	Big-scale sand smelt	E	1	+	Ath_boy
Barbatula barbatula	Stone loach	F	1	+	Bar_bar
Barbus barbus	Barbel	F	1	+	Bar_bab
Barbus meridionalis	Mediterranean barbel	F	1		

Species name	Common name	Species status	Species occurrence	Species occurrence in the TEF dataset	Detected MOTU's abbreviation name
Blicca bjoerkna	White bream	F	1	+	Bli_bjo
Carassius carassius	Crucian carp	F	1	+	
Carassius auratus	Goldfish	F	1	+	Car_spp
Carassius gibelio	Prussian carp	F	1	+	
Chelon labrosus	Thicklip grey mullet	E	1		Che_lab
Coregonus lavaretus	European whitefish	F	1	+	Cor_lav
Cottus gobio	Bulhead	F	1	+	Cot_sp
Chondrostoma nasus	Common nase	F	1	+	
Parachondrostoma toxostoma	South-west European nase	F	1	+	Cypr_1
Telestes souffia	Western verone	F	1	+	
Ctenopharyngodon idella	Grass carp	F	1		Cypr_2
Hypophthalmichthys molitrix	Silver carp	F	2	+	
Cyprinus carpio	Common carp	F	1	+	Cyp_car
Dicentrarchus labrax	European seabass	E	1	+	Dic_lab
Esox lucius	Northern pike	F	1	+	Eso_luc
Gambusia holbrooki	Mosquito fish	F	1	+	Gam_aff
Gasterosteus aculeatus	Three-spined stickleback	F	1	+	Gas_acu
Gobio gobio	Gudgeon	F	1	+	Gob_gob

Species name	Common name	Species status	Species occurrence	Species occurrence in the TEF dataset	Detected MOTU's abbreviation name
Gymnocephalus cernuus	Ruffe	F	1	+	Gym_cern
Lampetra planeri	European brook lamprey	F	1	+	Lam_spp
Lepomis gibbosus	Pumpkinseed	F	1	+	Lep_gib
Leucaspius delineatus	Sunbleak	F	1	+	Leu_del*
Leuciscus leuciscus	Dace	F	1	+	Leu_spp
Leuciscus idus	Ide	F	1	+	
Liza aurata	Golden grey mullet	E	1		
Liza ramada	Thinlip grey mullet	E	1	+	Liz_ram
Lota lota	Burbot	F	1	+	Lot_lot*
Micropterus salmoides	Largemouth blackbass	F	1	+	Mic_sal
Misgurnus fossilis	Weather loach	F	1		Mis_fos
Mugil cephalus	Flathead grey mullet	E	1		Mug_cep
Oncorhynchus mykiss	Rainbow trout	F	1		Onc_myk
Perca fluviatilis	Perch	F	1	+	Perc_flu
Petromyzon marinus	Sea lamprey	D	1		
Phoxinus phoxinus	Minnow	F	1	+	Pho_pho
Pseudorasbora parva	Topmouth gudgeon	F	1	+	Pse_par
Rhodeus sericeus	Bitterling	F	1	+	Rho_ser
Rutilus rutilus	Roach	F	1	+	Rut_rut

Species name	Common name	Species status	Species occurrence	Species occurrence in the TEF dataset	Detected MOTU's abbreviation name
Rutilus rutilus	Roach	F	1	+	Rut_rut
Salaria fluviatilis	Freshwater blenny	F	1	+	Sal_flu
Salmo salar	Atlantic salmon	D	3		Sal_sal
Salmo trutta	Brown trout	F	1	+	Sal_tru
Salvelinus umbla	Arctic Charr	F	2		Sal_spp
Salvelinus fontinalis	Brook trout	F	2		
Salvelinus namaycush	Salmon trout	F	2		
Sander lucioperca	Zander	F	1	+	San_luc
Scardinius erythrophthalmus	Rudd	F	1	+	Sca_ery
Silurus glanis	Wels catfish	F	1	+	Sil_gla
Squalius cephalus	Chub	F	1	+	Squ_cep
Thymallus thymallus	Grayling	F	1	+	Thy_thy
Tinca tinca	Tench	F	1	+	Tin_tin
Zingel asper	Apron	F	1	+	Zin_asp

Reference

Keith P., Persat H., Feunteun E. & Allardi (coords) (2011). Les poissons d'eau douce de France. Biotope, Mèze; Muséum national d’histoire naturelle, Paris (collection Inventaire et biodiversité). 552p.

Supplementary Table 3. Comparison of eDNA metabarcoding and traditional sampling methods on the Danube River.

The sampling site was located on the main channel of the Danube River 43 km downstream from Vienna (48° 9'39.61"N, 16°57'2.14"E). The water samples for eDNA analyses were collected on July 11, 2017, and the fish sampling session combining different sampling methods was performed from October 11 – 14, 2016. The water temperatures were 12.2 and 20.1°C, and the water conductivities were 475 and 339 µS in October 2016 and July 2017, respectively.

<u>eDNA metabarcoding method:</u>

Two water samples were collected by boat in the middle of the channel using a peristaltic pump (nominal flow of 1.67 L.min-1). The sampled water was filtered *in situ* through the VigiDNA® 0.45-µM capsule for 28 and 29 min. The eDNA metabarcoding analyses were carried out according to the same protocol as that used to monitor the Rhône River (see Methods), and a library was constructed and then sequenced in a Miseq 2500 lane.

<u>Traditional fish sampling methods:</u>

Electrofishing sampling utilized 31 strips collected during daylight (TEF-day, total of 6590 m) and 12 strips collected during the night (TEF-night, total of 1960 m) using an EFKO 11.00 KW DC generator with a 1.20-m-long floating copper cathode, a boom mounted anode (2.2 m width, approx. 580 V, 20 A) and a 3-m-long handheld anode (47 cm in diameter, approx. 580 V, 12 A) for the sampling of littoral habitats. The electrified benthic frame trawl consisted of a stainless-steel frame (2 m long and 1 m high) with a drifting net attached (5 m long, 10 mm mesh) and was equipped with wheels to hold the frame 6 cm from the bottom. The chassis was connected to an EFKO 11.00 KW DC generator. Trawling was done during the day for a total distance of approximately 1700 m three times. A total of 10 benthic 50-m-long lines with 50 baited hooks (hook sizes 1 to 12) exposed overnight in the middle of the river were used to sample the benthic fish assemblage. Drift nets were used to sample the deeper sections

in the middle of the river and gravel banks 20 to 50 m from the shoreline. A 3-layered trammel polyamide net with a mesh size of 200, 40 or 200 mm, a length of 25 m and a depth of 2 m was used. The average length of sampled stretches was approximately 500 m for a total of 3200 m.

Relative abundances of fish species caught by the different traditional fish sampling methods and of DNA copies from species detected from the eDNA samples. The total number of fish caught, total number of reads, total number of taxa detected by the different methods and the combination of all traditional sampling methods are also specified.

Species	Bottom trawl	Drift net	TEF-Night	TEF-Day	Longline	All traditional methods	eDNAm
Neogobius melanostomus	12.50%		37.50%	16.10%	73.00%	24.60%	41.90%
Alburnus alburnus			21.70%	61.00%		45.30%	2.30%
Neogobius gymnotrachelus							14.80%
Neogobius kessleri			2.90%	1.00%	7.90%	1.90%	13.40%
Barbus barbus	62.50%	65.40%	8.30%	4.00%	6.30%	6.80%	6.90%
Abramis brama	12.50%	15.40%	4.90%	2.00%	1.60%	3.10%	5.90%
Chondrostoma nasus	12.50%	19.20%	2.40%	3.50%		3.30%	2.30%
Ctenopharyngodon idella							2.10%
Squalius cephalus			2.20%	2.00%		1.90%	1.20%
Gymnocephalus schraetser			1.50%	0.90%	11.10%	1.50%	
Lota lota			2.90%	0.20%		1.00%	
Ballerus sapa			0.20%			0.10%	1.90%

Species	Bottom trawl	Drift net	TEF-Night	TEF-Day	Longline	All traditional methods	eDNAm
Vimba vimba			0.20%	0.70%		0.50%	
Blicca bjoerkna			4.40%	2.00%		2.60%	
Perca fluviatilis			0.70%	1.70%		1.30%	0.60%
Cottus gobio			1.70%	0.40%		0.80%	1.00%
Rutilus rutilus			1.20%	1.30%		1.20%	0.40%
Zingel zingel			0.70%	0.40%		0.50%	1.00%
Silurus glanis			1.50%	0.60%		0.80%	0.70%
Proterorhinus semilunaris				0.40%		0.30%	1.00%
Leuciscus idus			1.50%	0.30%		0.60%	0.30%
Aspius aspius			0.70%	0.40%		0.50%	0.40%
Sander lucioperca			0.70%			0.20%	0.60%
Cyprinus carpio			0.50%			0.10%	0.60%
Romanogobio vladykovi			0.70%	0.20%		0.40%	0.30%
Gymnocephalus cernuus				0.40%		0.30%	
Rutilus pigus			0.50%	0.20%		0.30%	
Gymnocephalus baloni							0.20%
Salmo trutta							0.20%
Sander volgensis			0.50%			0.10%	

Species	Bottom trawl	Drift net	TEF-Night	TEF-Day	Longline	All traditional methods	eDNAm
Salvelinus sp.							0.10%
Zingel streber				0.10%		0.10%	
Alburnoides bipunctatus							0.10%
Nb fish caught	8	26	411	897	63	1405	
Nb of reads							421237
Nb. of taxa	4	3	24	23	5	27	25

When the sampling effort was higher and when complementary fishing methods were used in addition to TEF, the number of species caught was comparable to the number of species detected by eDNA metabarcoding with our workflow. The relative abundance of benthic species in eDNA samples (e.g., *Neogobio* spp.) was higher than that in TEF samples (55.3% versus 17.1%) and closer to the values obtained by longline fishing (81%). By contrast, the relative abundance of bleak (*Alburnus alburnus*) was higher in electrofishing samples than in eDNAm samples.

Supplementary Table 4. Literature review of depositional velocity and transport distance of fine particulate organic matter (FPOM)in stream and river

Particle depositional velocity (V_{dep}) of (FPOM) and transport distance needed to retain 63.2% of FPOM in the riverbed (Sp) values obtained in previous experimental and observational studies (Cushing et al. 1993, Newbold et al. 2005, Ock et Takemon 2010, Paul et al. 2002, Thomas et al. 2001, Young et Jowett 2005). Physical characteristics of stream reaches used in the different studies (depth, width, velocity, discharge) and seston type: FPOM-r: fine particulate organic matter radio labeled (53 – 106 µm), MPOM-r: large particulate organic matter radio labeled (107 - 250 µm), VFPOM-r very fine particulate organic matter radio-labeled (16 - 52 µm), FLB fluorescently labeled bacteria (2 µm), FLY fluorescently labeled yeast (5 – 7 µm), DPO derived plankton observation in the outlet of a lake, Diatom-r diatom radio labeled, na: data not available. Values in italics are calculated from other hydraulic parameters. The values obtained by Minshall et al. (2000) are included in Thomas et al. (2001).

Reference	Seston type	Depth (m)	Width (m)	Waterflow ($m^3.s^{-1}$)	Velocity ($m.s^{-1}$)	V_{dep} ($mm.s^{-1}$)	Sp (m)
Cushing et al. (1993)	FPOM-r	0.34	7.10	0.67	0.27	0.12	800
Cushing et al. (1993)	FPOM-r	0.14	5.80	0.23	0.29	0.07	630
Cushing et al. (1993)	FPOM-r	0.34	7.10	0.63	0.27	0.16	580
Hall et al. (1996)	FLB	na	45.00	0.00495	na	0.093	80
Newbold et al. (2005)	VFPOM-r	0.31	*2.52*	0.225	0.29	0.12	750
Newbold et al. (2005)	FPOM-r	0.31	*2.52*	0.225	0.29	0.18	510
Ock et Takemon (2010)	DPO	0.27	3.38	0.29	0.42	0.053	2200
Ock et Takemon (2010)	DPO	0.54	111.54	33.6	2.08	0.286	3910
Ock et Takemon (2010)	DPO	1.67	14.58	10.1	0.5	0.044	19190
Ock et Takemon (2010)	DPO	1.7	90.89	130	0.92	0.247	6310

Reference	Seston type	Depth (m)	Width (m)	Waterflow ($m^3.s^{-1}$)	Velocity ($m.s^{-1}$)	V_{dep} ($mm.s^{-1}$)	Sp (m)
Paul et Hall (2002)	FLB	0.036	1.15	0.0002	0.0048	0.044	4
Paul et Hall (2002)	FLB	0.032	1.97	0.0004	0.0064	0.041	5
Paul et Hall (2002)	FLB	0.083	2.32	0.0029	0.0151	0.052	24
Paul et Hall (2002)	FLB	0.076	1.96	0.0057	0.0383	0.039	74
Paul et Hall (2002)	FLB	0.085	7.33	0.053	0.0854	0.043	168
Paul et Hall (2002)	FLB	0.143	9.38	0.12	0.0894	0.031	409
Miller et Georgian (1992) *	pollen	na	na	na	na	0.21	190
Miller et Georgian (1992) *	pollen	na	na	na	na	0.25	122
Thomas et al. (2001)	VFPOM-r	0.33	*5.55*	0.897	0.49	0.13	1153
Thomas et al. (2001)	FPOM-r	0.33	*5.55*	0.897	0.49	0.34	416
Thomas et al. (2001)	VFPOM-r	0.06	*1.67*	0.013	0.13	0.43	15.2
Thomas et al. (2001)	FPOM-r	0.06	*1.67*	0.013	0.13	0.62	9.8
Thomas et al. (2001)	VFPOM-r	0.06	*1.67*	0.013	0.13	0.42	23.3
Thomas et al. (2001)	FPOM-r	0.06	*1.67*	0.013	0.13	1.03	8.8
Thomas et al. (2001)	VFPOM-r	0.31	*2.50*	0.225	0.29	0.11	843
Thomas et al. (2001)	FPOM-r	0.31	*2.50*	0.225	0.29	0.17	526
Thomas et al. (2001)	FPOM-r	0.26	*1.35*	0.077	0.22	0.24	238
Thomas et al. (2001)	VFPOM-r	0.26	*1.35*	0.077	0.22	0.16	308
Thomas et al. (2001)	VFPOM-r	0.09	*0.63*	0.004	0.07	0.43	13.4
Thomas et al. (2001)	FPOM-r	0.09	*0.63*	0.004	0.07	0.63	9
Thomas et al. (2001)	MFPOM-r	0.09	*0.63*	0.004	0.07	0.53	94.4

Reference	Seston type	Depth (m)	Width (m)	Waterflow ($m^3.s^{-1}$)	Velocity ($m.s^{-1}$)	V_{dep} ($mm.s^{-1}$)	Sp (m)
Thomas et al. (2001)	Diatom	0.09	*0.63*	0.004	0.07	0.99	4.8
Thomas et al. (2001)	VFPOM-r	0.09	*0.63*	0.004	0.07	1.09	4.4
Thomas et al. (2001)	FPOM-r	0.09	*0.63*	0.004	0.07	1.1	4.3
Young et Jowett (2005)	DPO	1.738	*97.46*	145	0.856	0.46	3224
Median value		0.1415	2.50	0.077	0.175	0.18	190
First Quartile		0.0845	1.35	0.004	0.07	0.0815	14.3
Third Quartile		0.315	7.10	0.29	0.29	0.43	690
Minimum value		0.032	0.63	0.0002	0.0048	0.031	4
Maximum value		1.738	111.54	145	2.08	1.1	19190

References

Cushing, C. E., Minshall, G. W., Newbold, J. D. Transport dynamics of fine particulate organic matter in two Idaho streams. Limnol. Oceanogr. 38, 1101-1115 (1993).

Minshall, G. W., Thomas, S. A., Newbold, J. D., Monaghan, M. T. & Cushing, C. E. Physical factors influencing fine organic particle transport and deposition in streams. J. N. Am. Benthol. Soc. 19, 1-16 (2000).

Newbold, J. D., Thomas, S. A., Minshall, G. W., Cushing, C. E. & Georgian, T. Deposition, benthic residence, and resuspension of fine organic particles in a mountain stream. Limnol. Oceanogr. 50(5), 1571–1580 (2005).

Ock, G. & Takemon, Y. Estimation of transport distance of fine particulate organic matter in relation to channel morphology in tailwaters of the Lake Biwa and reservoir dams. Landscape Ecol. Eng., 161–169 (2010).

Paul, M. J. & Hall, R. O. Particle transport and transient storage along a stream-size gradient in the Hubbard Brook Experimental Forest. J. N. Am. Benthol. Soc. 21, 195-205 (2002).

Thomas, S. A., Newbold, J. D., Monaghan, M. T., Minshall, G. M., Georgian, T. & Cushing, C. E. The influence of particle size on seston deposition in streams. Limnol. Oceanogr. 46, 1415-1424 (2001).

Young, R. & Jowett, I. Observations and modelling of seston transport in the Waitaki River downstream of the Waitaki Dam. (Report No. 1030, Cawthron Institute, 2005).

Supplementary Figure 1. Influence of sampling effort on eDNA metabarcoding detection efficiency

Ten water samples were collected in the Rhône main channel (KP 126) on 30/09/2015 to evaluate the influence of the sampling effort on the detection efficiency. The filtration device was lowered from the center of a bridge, and the water was filtered through the VigiDNA® 0.45-µM capsule for 30 min. eDNA analyses were performed like those for the other samples, and a library was constructed and then sequenced in a Miseq 2500 lane. The numbers of species per sample varied between 26 and 30, and the species richness estimated by the Chao1 index (Chao 1984) was 30. Only five species' MOTUs with relative abundances lower than 3‰ were not represented in all 10 water samples.

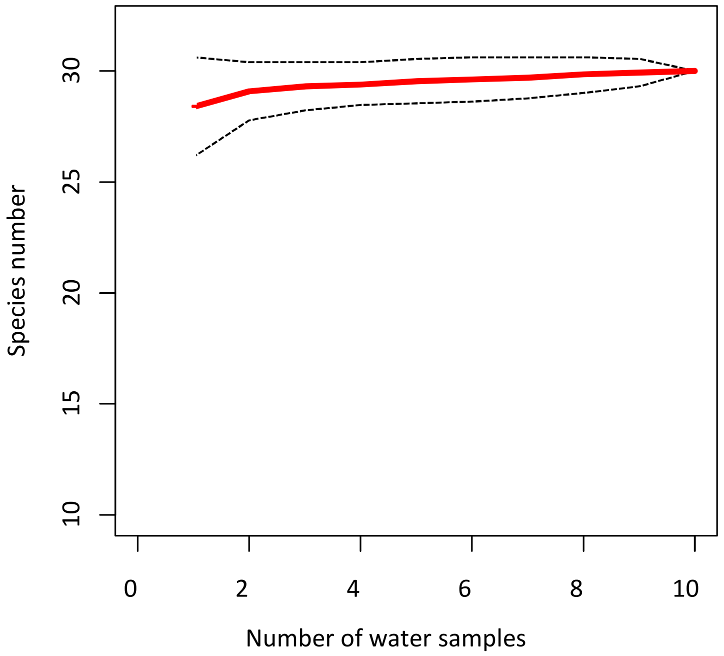

Species accumulation curve of fish assemblages observed in ten eDNA samples

A species accumulation curve (*speccacum* function from R package *vegan*) showed that two samples were sufficient to detect 96.9% (29 species) of the total number of species. Non-parametric Spearman's correlation coefficients (with Bonferroni correction for multiple comparisons) between the relative abundance distributions of species in the ten samples ranged between 0.851 and 0.970

(mean = 0.916, all p-values < 10^{-6}). The range of Spearman's correlation coefficients between all combinations of sample pairs was narrowed towards higher values (0.928 to 0.998, all p-values < 10^{-6}, mean value = 0.973). Two samples were sufficient to comprehensively evaluate the number of species with more than 95% of the species richness, which represents a higher efficiency than those obtained in lake (Hänfling et al. 2016) or coral reef (Yamamoto et al. 2017) studies.

References

Hänfling B., Handley, L. L. Read, D. S.,Hahn, C., Li, J., Nichols, P., Blackman, R. C., Oliver, A. & Winfield, I. J. Environmental DNA metabarcoding of lake fish communities reflects long-term data from established survey methods. *Mol. Ecol.* 25, 3101–3119 (2016).

Yamamoto S., Masuda, R., Sato, Y., Sado, T., Araki, H., Kondoh, M., Minamoto, T. & Miya, M. Environmental DNA metabarcoding reveals local fish communities in a species-rich coastal sea. *Sci. Rep.-UK* 7, 40368, DOI: 10.1038/srep40368 (2017).

Supplementary Figure 2. Longitudinal profile of MOTUs and species abundances along the Rhône river.

Longitudinal profile of species abundances along the main channel of the Rhône River as revealed by eDNA metabarcoding (red line, in standardized number of reads, left Y axis) and TEF (blue line, in catch per unit effort CPUE, right Y axis). The MOTUs Cypr_1 and Cypr_2 detected three species (*Chondrostoma nasus*, *Parachondrostoma toxostoma, Telestes souffia)* and two species (*Leuciscus, Leuciscus idus*), respectively (See explanations in the text).

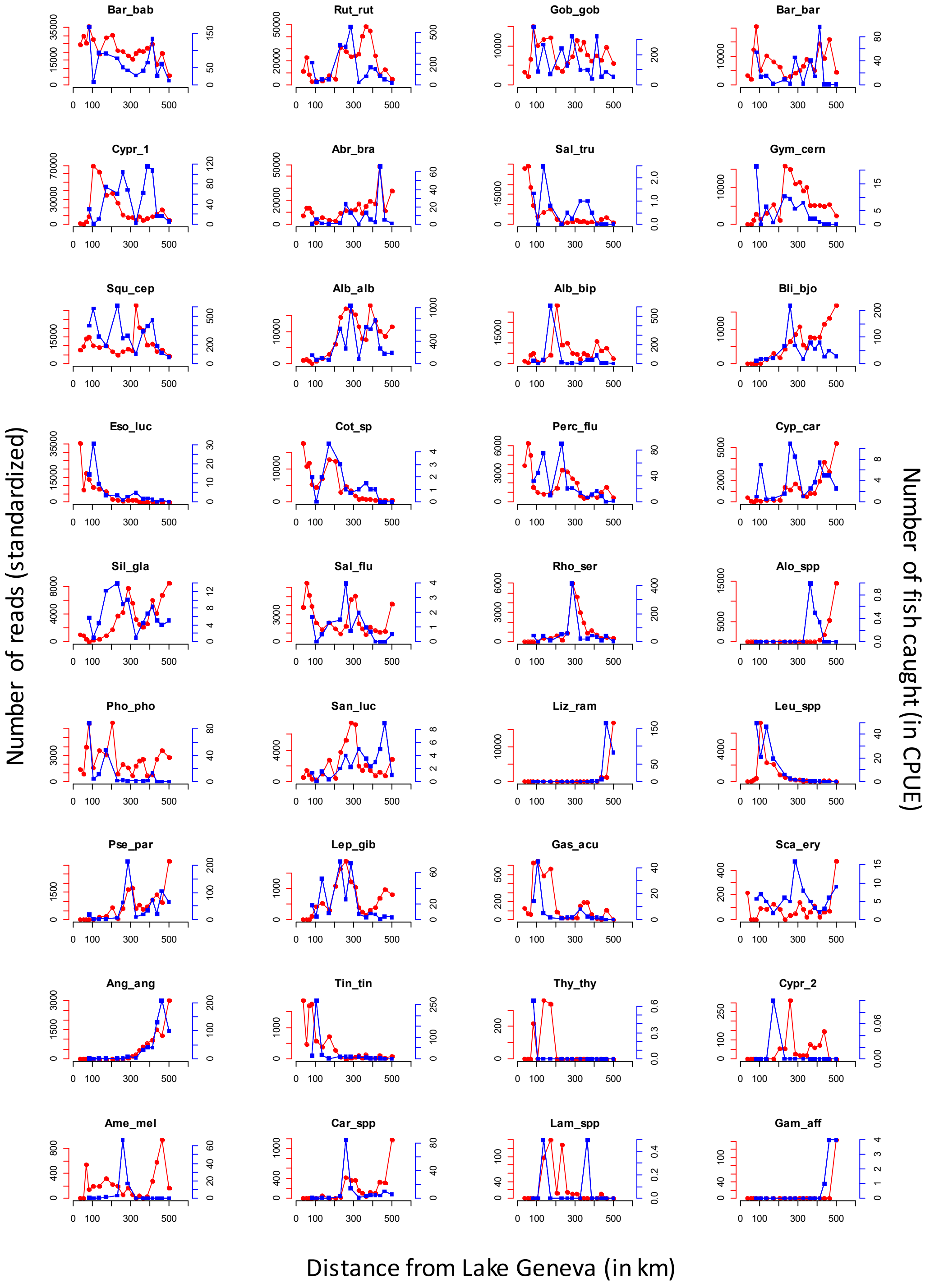

Supplementary Figure 3. Simulated maximal detection distance of eDNA

Simulated maximal detection distance (in km) of eDNA as a function of water velocity (u in $m.s^{-1}$) and river depth (h in m) for an initial released quantity of 2000 mtDNA copies / L multiplied by 2.5, to simulate taking a 2.5-L sample (Wilcox et al. 2016). The predicted maximal detection distance is defined as the distance for which less than one mtDNA copy of a hypothetical MOTU quantity released upstream is still present in the water column. The value of the deposition velocity coefficient V_{dep} used in the model is the median value of previous published estimations of the fine particulate organic matter (FPOM) transfer from the water column to the riverbed (see text for explanation and Suppl. Table 4).

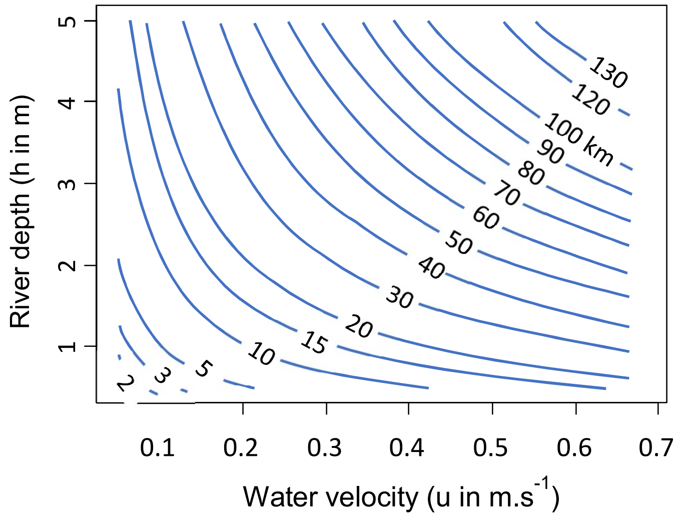

Reference

Wilcox, T. M., McKelvey, K. S., Young, M. K., Sepulveda, A. J., Shepard, B. B., Jane, S. F. & Schwartz, M. K. Understanding environmental DNA detection probabilities: a case study using a stream-dwelling char Salvelinus fontinalis. *Biol. Conserv.* 194, 209–216 (2016).

Supplementary Figure 4. Water filtration device sampler and cross-flow filtration capsule used to sample eDNA

Water filtration device sampler (Photo A) used in this work to sample eDNA from a boat (Photo B) or a bridge (Photo C). At the end of each filtration (30 min for a water volume of approximately 30 L), the water inside the VigiDNA® 0.45-µM cross-flowfiltration capsule (SPYGEN, le Bourget du Lac, France) was emptied, and the capsule was filled with 80 mL of CL1 Conservation buffer (SPYGEN, le Bourget du Lac, France) and stored at room temperature before DNA extraction (Photos M. Rocle (Compagnie Nationale du Rhône, Direction de l'Ingénierie, Lyon, France).

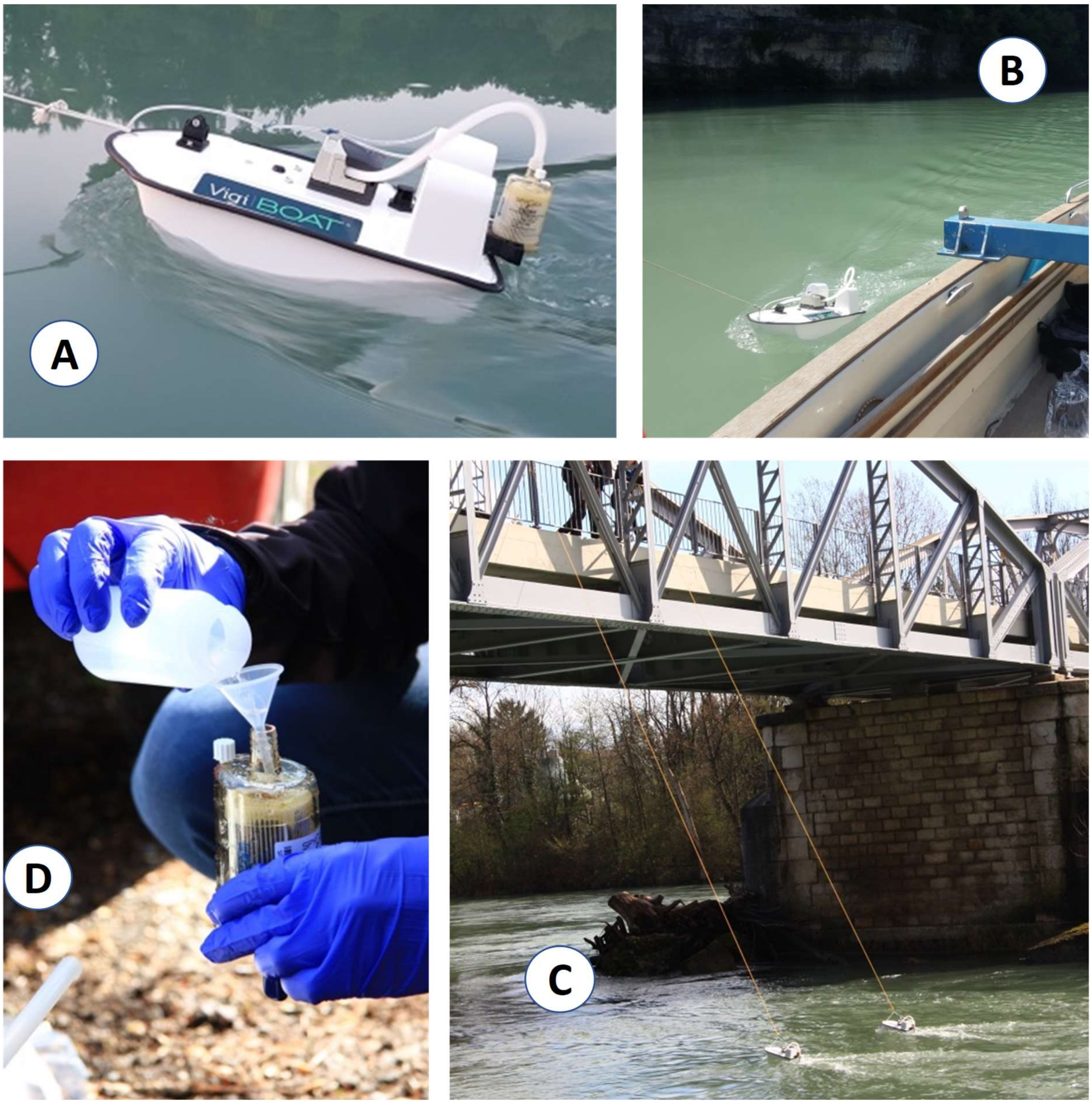

Supplement: Supplementary file 1 — Supplementary Material [file 41598_2018_28424_MOESM1_ESM.pdf]
